# Reversible Dimerization of Cytochrome Oxidase in Detergent Solution as Detected by Dynamic Light Scattering

**DOI:** 10.3390/ijms27198744

**Published:** 2026-09-30

**Authors:** Natalia V. Azarkina, Victor N. Orlov, Maria S. Muntyan, Yury M. Chesnokov, Semen V. Nesterov, Ilya P. Oleynikov, Tatiana V. Vygodina

**Affiliations:** 1 A.N. Belozersky Institute of Physico-Chemical Biology, M.V. Lomonosov Moscow State University, 119992 Moscow, Russia; orlovvn@belozersky.msu.ru (V.N.O.); muntyan@genebee.msu.ru (M.S.M.);; 2 Kurchatov Complex of NBICS-Technologies, National Research Center “Kurchatov Institute”, 123182 Moscow, Russia

**Keywords:** cytochrome *c* oxidase, dynamic light scattering, dimerization, monomerization, amphipol

## Abstract

Reversible changes in the quaternary structure of eukaryotic cytochrome *c* oxidase (CcO) are presumably involved in the enzyme activity regulation. Conventional methods for determining quaternary states are relatively complex. We applied dynamic light scattering (DLS) to estimate the size of CcO in micelles and identified different oligomeric forms in dynamic equilibrium. Thin-layer gel filtration and non-denaturing gel electrophoresis revealed CcO forms with different molecular masses. CcO quaternary structure was analyzed by cryo-electron microscopy. Prokaryotic cytochrome oxidase served as a control. Increasing protein concentration, cholate treatment, and acidification shifted the equilibrium toward larger micellar structures, suggesting CcO oligomerization. Protein dilution, dodecyl maltoside at high concentrations, alkalinization, and treatment with Triton X-100 reversed this process. The apparent size of prokaryotic cytochrome oxidase micelles remained constant regardless of enzyme concentration and presence of cholate. The apparent molecular masses of CcO in diluted and concentrated preparations corresponded to monomers and dimers, respectively. The amphipol-stabilized CcO forms, both monomeric and the cholate-containing dimeric, retained their characteristic sizes across various enzyme concentrations. Given that the DLS-based identification of the CcO oligomeric states was confirmed by three independent methods mentioned above, DLS appears to be a convenient tool for characterizing CcO quaternary structures in solution and monitoring their interconversion.

## 1. Introduction

Cytochrome *c* oxidase (CcO) is a key enzyme of the electron transport chain, located in the inner mitochondrial membrane, where it catalyzes the final step of aerobic respiration. CcO catalyzes the four-electron reduction in molecular oxygen to form two water molecules. The direct electron donor for CcO is soluble cytochrome *c*, which interacts with the enzyme on its external surface, on the intermembrane-space side. During the oxidase reaction, “chemical” protons involved in H_2_O formation are captured from the mitochondrial internal aqueous phase, i.e., the mitochondrial matrix. In addition, oxygen reduction drives proton pumping from the mitochondrial matrix to the intermembrane space. Thus, the catalytic activity of CcO is coupled to the generation of a proton-motive force (ΔµH^+^) across the membrane (see recent reviews [1,2,3]). Heme-copper terminal oxidases localized in the plasma membrane of aerobic bacteria function in a similar manner. They typically consist of three to four subunits that are homologous to the core subunits of mitochondrial CcO [4,5]. One of the most extensively studied bacterial oxidases is the *aa*_3_-type cytochrome *c* oxidase from *Rhodobacter sphaeroides* [6,7].

Early studies noted that, upon solubilization, mitochondrial CcO exists in two forms: monomeric and dimeric [8]. The relative abundance of these forms depended on the isolation conditions, particularly on the detergents used [9,10,11]. Initial isolation methods used cholate and deoxycholate, enabling the stable multi-subunit enzyme to be obtained in good yield [12,13]. It was soon established that bile salts inhibit CcO [14,15]; however, the enzyme activity could be partially recovered by replacing these detergents with non-ionic alternatives [16]. Because of this, the use of cholate at the initial stage of isolation, followed by non-ionic detergents at the final stages, has become widespread [8,17]. Later, an alternative procedure was developed using exclusively non-ionic detergents such as DM or Triton X-100 [18]. In some laboratories, CcO is still isolated using bile salts [19,20], after which the enzyme is activated by dilution in an assay medium containing DM [21]. 

**Figure 1:**
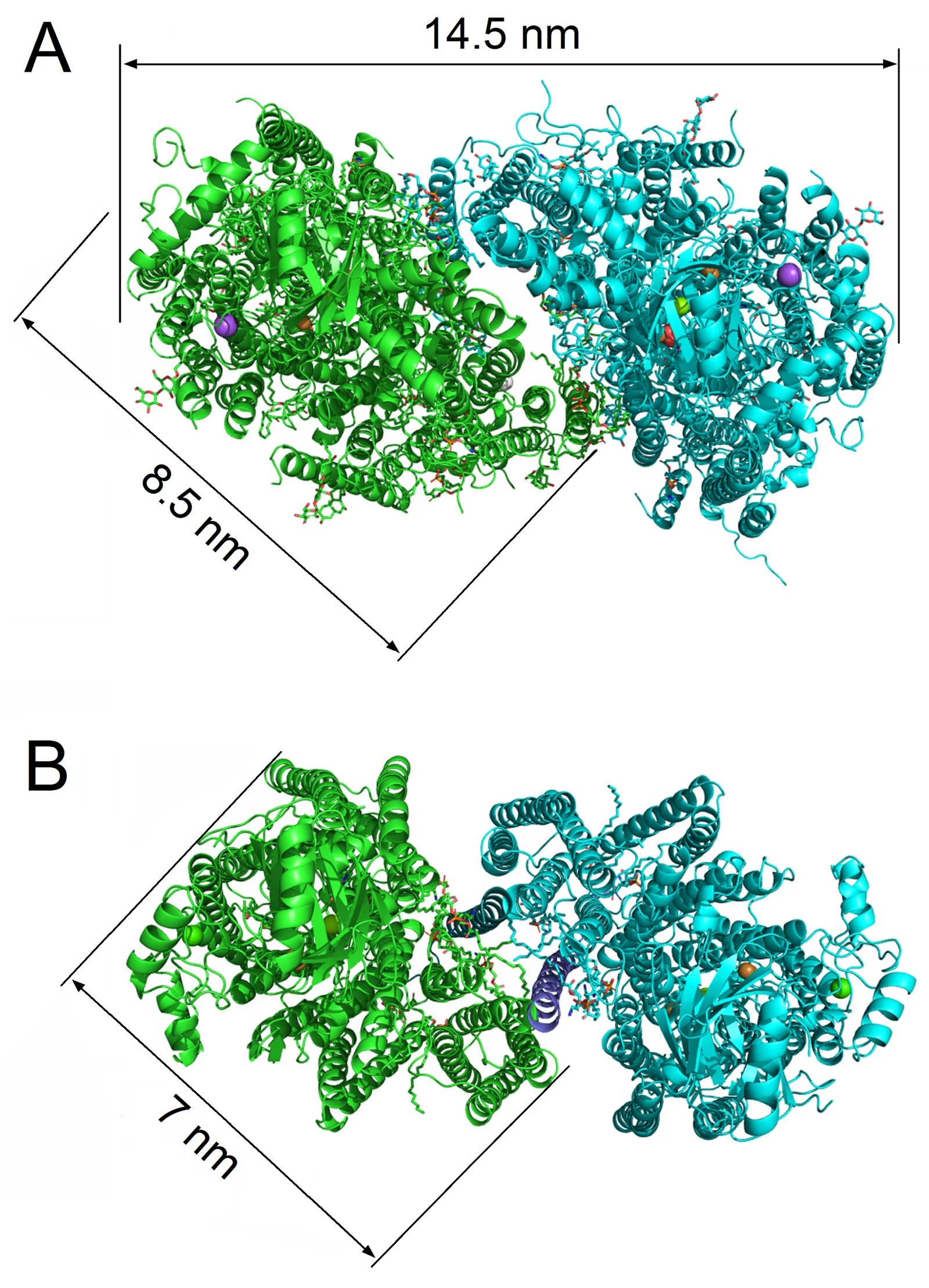
Three-dimensional structures of eukaryotic and prokaryotic cytochrome oxidases. View from the intermembrane space. (**A**) Dimer of cytochrome *c* oxidase (CcO) from bovine heart mitochondria (PDB 5b1a). Different monomers are highlighted in green and blue. (**B**) Cytochrome oxidase of the *aa*_3_-type from *Rhodobacter sphaeroides* (PDB 1m56). The two monomers (green and blue) are aligned with the structure of the mitochondrial dimer (panel (**A**)). Subunit IV of each monomer is highlighted in a darker color.

Since high cholate concentrations inhibit CcO, the cholate concentration should be lowered at the final stage of isolation. In addition to inhibition, cholate exerts a further effect on the enzyme by causing its dimerization [22,23]. One potential mechanism underlying the latter is that cholate molecules exhibit structural and chemical similarity to an as-yet unidentified physiological ligand (possibly ADP) that induces the reversible dimerization of CcO. Cholate molecules involved in dimerization bind to the enzyme with high affinity. Consequently, preparations of mitochondrial CcO isolated by the standard method—and therefore inevitably containing residual cholate—appear to predominantly contain enzyme dimers. For this reason, until recently, all resolved three-dimensional X-ray structures of eukaryotic CcO revealed the dimeric form of the enzyme [24,25]. In contrast, prokaryotic heme-copper terminal oxidases are mostly observed as monomers in the X-ray structures [6,26,27,28]. Despite the general tendency toward a monomeric state among prokaryotic heme-copper terminal oxidases, recent low-temperature electron microscopy data demonstrated that the *bo*_3_-type terminal oxidase from *Escherichia coli* is able to form a dimer [29]. Figure 1 illustrates representative three-dimensional structures of cytochrome oxidase, exemplified by the mitochondrial CcO dimer (panel A) and the *aa*_3_-type cytochrome oxidase from *R. sphaeroides* (panel B). For comparative clarity, the second case illustrates two protein molecules aligned in the same orientation as the two monomers in panel A. Notably, in the bacterial enzyme, the formation of the interface between monomers is sterically hampered by the α-helices of subunit IV.

It has long been noted that a number of factors (protein dilution, alkaline pH, non-ionic detergents) promote the transition of solubilized mitochondrial CcO to the monomeric state [30]. Musatov’s group studied in detail the reversible transition between the monomeric and dimeric forms and determined the exact conditions that support one or another quaternary structure [22]. To induce dimerization of solubilized CcO, it is sufficient to add cholate to the medium in an amount significantly exceeding that of the non-ionic detergent. Reducing the cholate concentration to a critical threshold initiates monomerization. In the absence of cholate, the enzyme exists entirely in its monomeric form regardless of other conditions such as protein concentration and pH. In general, there is a dynamic equilibrium in a CcO solution between dimeric and monomeric forms. The equilibrium is shifted by various factors—mainly protein and detergent concentrations, pH, and also the presence of specific agents. Among these, the strong non-ionic detergent Triton X-100 is particularly noteworthy. An effective and long-developed method for rapid and complete CcO monomerization is treatment with high concentrations of Triton X-100 (tens to hundreds of millimolar) in an alkaline medium [31,32,33]. The resulting preparation is homogeneous, stable, and has a normal optical spectrum but completely lacks catalytic activity, which, however, can be partially restored by replacing the detergent [34]. 

The state in which CcO functions *in vivo* remains a subject of debate [23]. It is currently widely accepted that both monomers and dimers are functional physiological forms, and that the reversible dimerization of CcO plays a key role in the regulation of the respiratory chain as a whole [35,36,37]. It is noteworthy that in preparations obtained using digitonin, CcO was identified in another state as a component of the respirosome complex. Such a complex usually includes, in addition to the CcO monomer, a monomer of respiratory complex I and a dimer of respiratory complex *bc*_1_ (see, for example, [38,39,40]). However, the ratio of components *in vivo* has not been unambiguously established (see the discussion of the issue in [23]). Notably, respirosomes incorporate the NDUFA4 CcO subunit, which is typical of the monomeric state of the enzyme [41] and is hypothesized to prevent dimerization [37]. The hypothesis of respirosomal organization of the respiratory chain is quite well-reasoned. Indeed, direct contact between some enzymes could lead to an acceleration of electron transfer overall. In some cases, the formation of respirosomes has been shown to correlate with decreased production of reactive oxygen species during respiration [40]. However, it cannot yet be definitively ruled out that respirosomes are an isolation artifact. 

Quite recently, the Shinzawa-Itoh group was the first to obtain the three-dimensional X-ray structure of the mitochondrial CcO in a monomeric form, which is in many ways different from the previously known dimeric one [42,43]. Furthermore, the authors successfully stabilized and investigated various quaternary forms of CcO in solution. To this end, the protein was stabilized by the amphipathic polymer amphipol. Amphipols consist of a hydrophobic homopolymer backbone with amphiphilic groups attached, which interact with the target protein and stabilize it. This new class of compounds has found wide application in the solubilization of membrane proteins (see reviews [44,45]). As reported by Shinzawa-Itoh et al. [42], amphipol A8-35 not only ensured the solubility of CcO but also prevented the interaction of protein molecules with each other. As a result, the catalytic activity of both quaternary forms was measured separately for the first time (for the monomer, at least under those conditions, it was about twice as high).

Apparently, the properties of CcO possessing distinct quaternary structures exhibit significant variations. These differences are of importance both in themselves and because they may determine the specific behavior of the enzyme under experimental conditions. Therefore, it appears expedient to develop a reliable and rapid method for determining the state of the enzyme in the preparation under study. The most commonly used methods for determining the quaternary structure of CcO include non-denaturing gel electrophoresis [42], gel filtration [11], and analytical ultracentrifugation [8]. Unfortunately, all of them are rather complex procedures, and the second and third methods also involve significant sample consumption. DLS is a technique based on particle size determination in a liquid sample. It seems to be an extremely attractive way to distinguish quaternary states of solubilized proteins. The data presented in this study indicate that DLS is indeed able to distinguish between the monomeric and dimeric forms of solubilized CcO, and to record their interconversions.

## 2. Results

### 2.1. Studies Based on the Dynamic Light Scattering (DLS) Method

DLS analysis of CcO in detergent micelles reveals particle size distribution. The method is not strictly quantitative because it does not account for the actual shape of the particles. Experimental data are presented as the result of the movement of ideal spheres. The output shows the contribution of “spheres” of different diameters to the total volume. The contribution of particles related to diameter A±x is determined by the integral of the distribution function in the vicinity of the maximum A.

#### 2.1.1. Size Distribution of CcO in Dodecyl Maltoside (DM) Micelles

**Figure 2:**
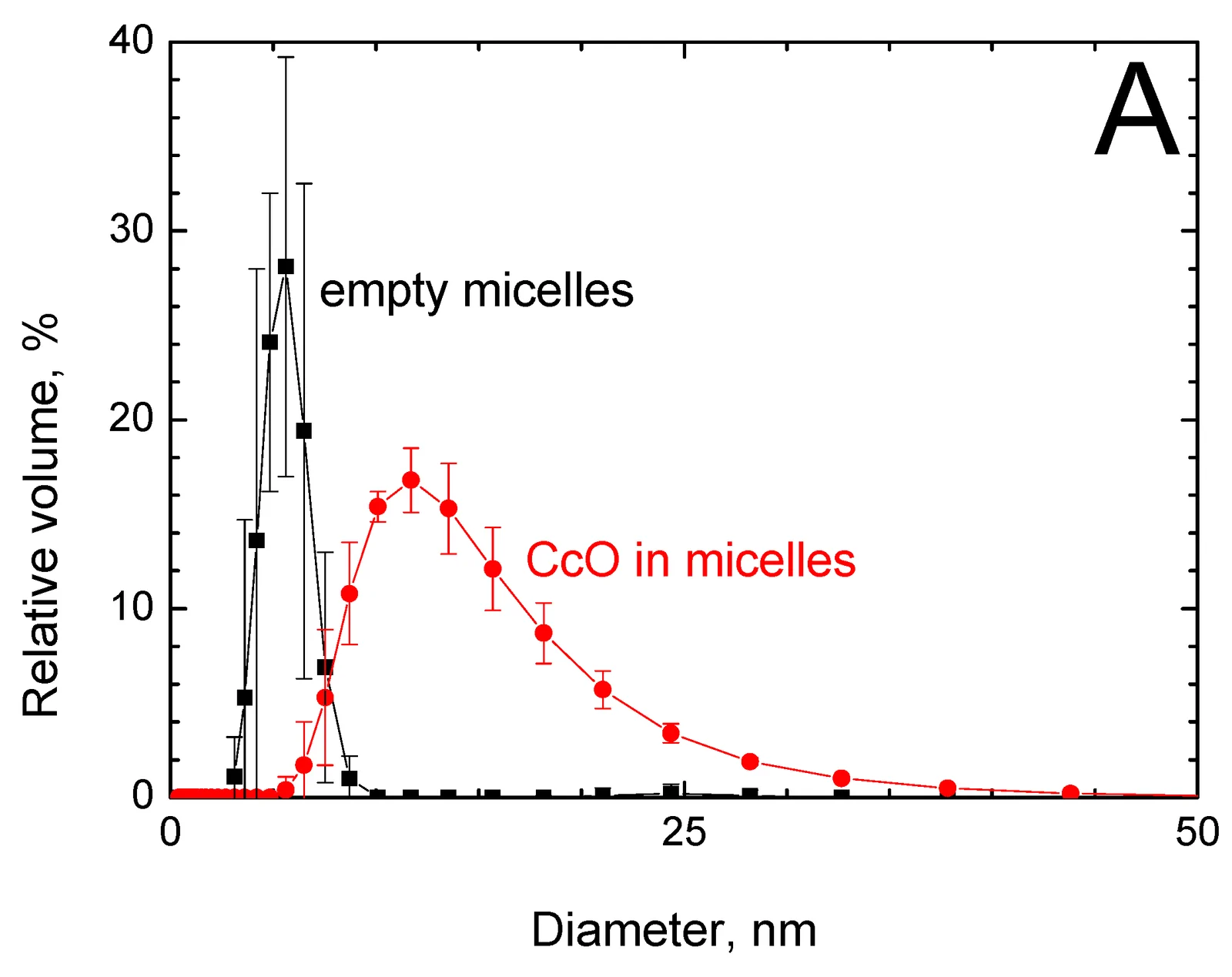

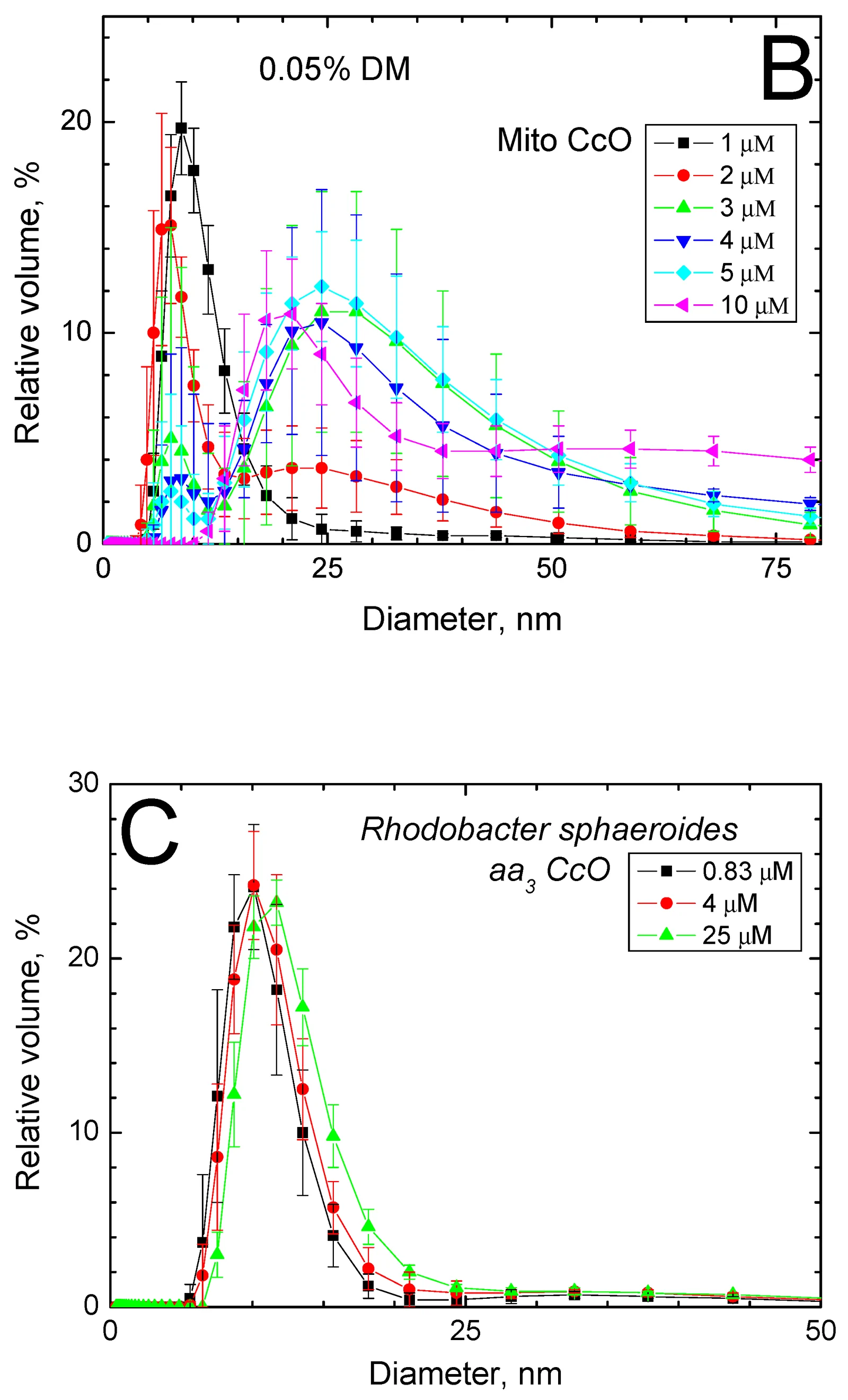
Micelle size distribution in a solution containing dodecyl maltoside (DM) and CcO. Experimental medium: 50 mM potassium phosphate, pH 7.4, 0.05% DM. From here on, the symbols represent average values and are presented with standard deviations. For clarity, they are connected by curves of the corresponding colors. Panels (**A**–**C**) show the results of representative experiments. Each experiment was repeated at least three times using at least two independent enzyme preparations. (**A**) Distribution of DM micelles in the absence of protein (black squares) and after the addition of 4 µM mitochondrial CcO (red circles). During the experiment, the count rate in the second case was approximately 30 times higher than in the first. (**B**) Distribution of micelles in the presence of different concentrations of mitochondrial CcO: 1 µM (black squares), 2 µM (red circles), 3 µM (green triangles), 4 µM (blue triangles), 5 µM (light blue diamonds), 10 µM (purple triangles). (**C**) Distribution of micelles in the presence of different concentrations of cytochrome oxidase *aa*_3_ from *R. sphaeroides*: 0.83 µM (black squares), 4 µM (red circles), 25 µM (green triangles).

The first sample was an aqueous solution of DM with a concentration of 0.05% (1 mM), which is 6.5 times higher than the critical micelle concentration (CMC) and is standard in most studies on solubilized CcO. The particle size distribution is represented by the black curve in Figure 2A. It appears as a symmetrical maximum centered around 5 nm and with a half-width of 2.2 nm. The obtained estimate of the diameter of a conventional sphere simulating a detergent micelle is in very good agreement with the literature data. Indeed, according to the results of small-angle X-ray scattering, the DM micelle in an aqueous environment is an ellipsoid with axes of about 2 and less than 6 nm [46]. After adding solubilized CcO to the DM solution (red curve), the response associated with empty detergent micelles is no longer detected. This can be explained by the sharp increase in the measured signal (the count rate in the sample after adding the protein is more than an order of magnitude higher compared with the initial one; see the figure caption). The broad asymmetric maximum on the red curve indicates the presence of particles with a nominal diameter significantly exceeding 5 nm. Clearly, the observed response is now determined by protein-containing micelles. Our further experiments will focus on assessing the size of such micelles.

It should be noted that resolution of the maxima (and, accordingly, the exact position of their centers) varied slightly between CcO preparations. In addition, a shift in the maximum within a few nm was sometimes observed over time in the same sample (cf. black and red curves in Supplementary Materials Figure S1), which may be due to aggregation and partial precipitation of micelles. The distribution shape was not affected by the ionic strength of the medium (compare the red curve with the green one in Figure S1, obtained after adding 100 mM KCl), which likely indicates the predominantly hydrophobic nature of the interaction between the particles.

In CcO samples well equilibrated under measurement conditions, the resolution of the maxima increased. The data obtained for different concentrations of CcO after 30 min preincubation (0.05% DM, pH 7.4, +25 °C) are shown in Figure 2B. At the lowest concentration of CcO (1 μM), the distribution maximum is in the range of 8–10 nm. The distribution is asymmetrical: particles with a diameter of less than 5 nm are practically absent, while those with a diameter of 15 nm make up about 20% of the total volume. With an increase in CcO concentration, the distribution maximum at 8–10 nm decreases in amplitude, which is accompanied by the appearance and growth of a new, wider maximum centered at 22–24 nm. The ratios of the areas under the distribution function in the region of 8–10 nm vs. 22–24 nm are approximately 3:1 for 2 µM CcO, 1:4 for 3 µM CcO, 1:7 for 4 µM CcO, and 1:10 for 5 µM CcO. At 10 µM CcO, the 8–10 nm maximum is virtually absent, while the position of the second one shifts to 20 nm. Additionally, very broad local maxima begin to appear at tens and hundreds of nm, which apparently indicates nonspecific aggregation.

Data from an experiment similar to Figure 2B but carried out at a higher detergent concentration (0.2% = 4 mM DM) are given in the Supplementary Materials (Figure S2A). Under these conditions, the distribution at 1 μM CcO exhibits a single maximum near 8 nm as previously. However, with increasing protein concentration, instead of a second maximum appearing, a shift in the single one to the right is observed, reaching a final position of 16–18 nm (at CcO concentrations greater than 20 μM). After incubation of CcO in the measurement medium for an hour, the distribution takes the shape with two maxima at 8 and 22 nm, similar to that observed at a lower DM concentration (blue curve in Figure S2A, cf. Figure 2B). Figure S2B demonstrates the particle size distributions at three different DM concentrations, at a fixed CcO concentration. In all cases, the distribution shows two maxima: the first, narrower, at 7–9 nm, and the second, wider, at 22–25 nm. An increase in DM concentration is accompanied by a growth of the first maximum and a gradual disappearance of the second. 

The micelle size distribution was also studied on cytochrome oxidase *aa*_3_ from *R. sphaeroides* (Figure 2C). A comparison of panels B and C shows that, unlike mitochondrial CcO, the concentration of the bacterial enzyme has virtually no effect on the distribution curve, which in all cases demonstrates a single maximum in the region of 10 nm. 

#### 2.1.2. Changes in CcO Quaternary Structure Are Reflected in the Size of Micelles

**Figure 3:**
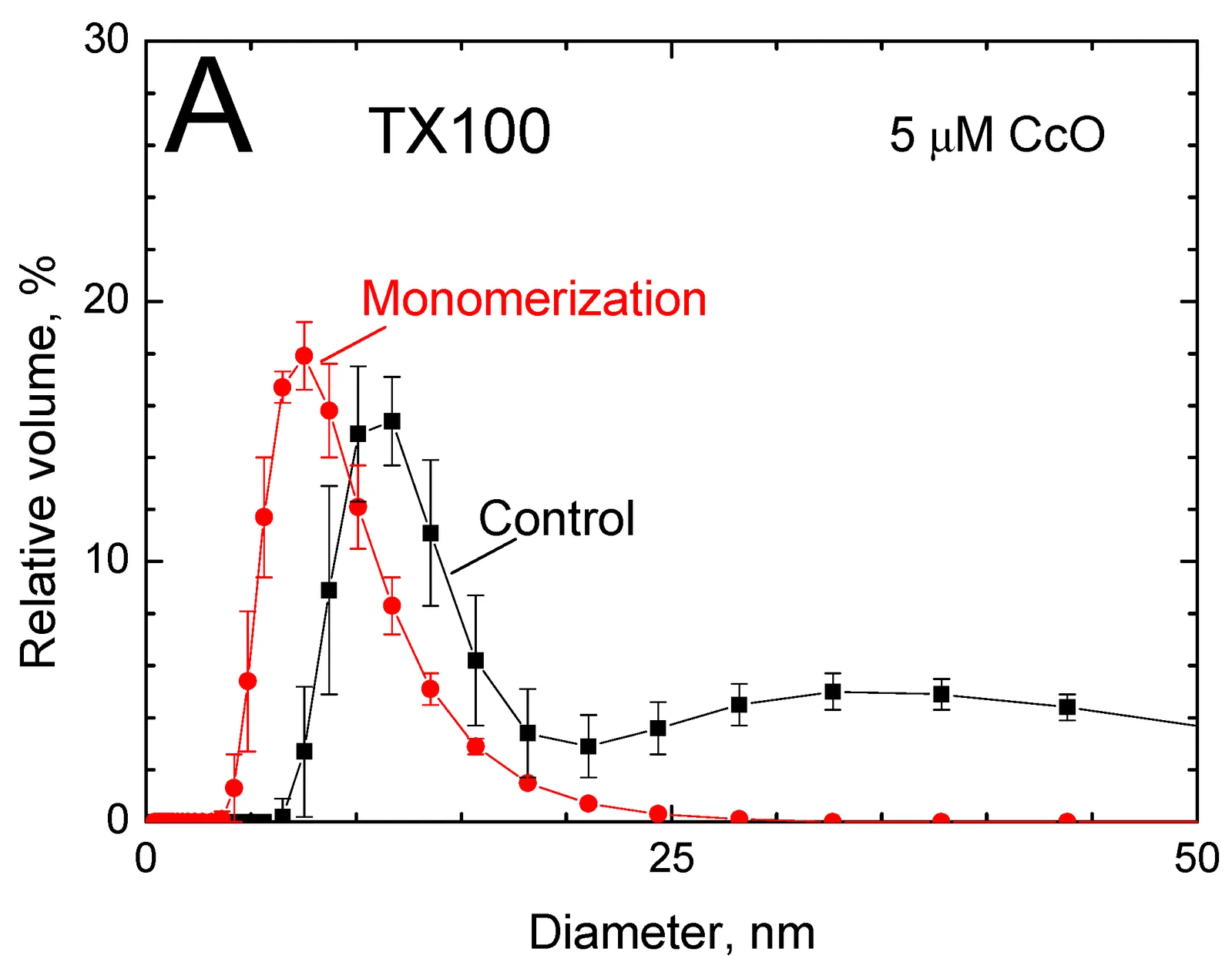

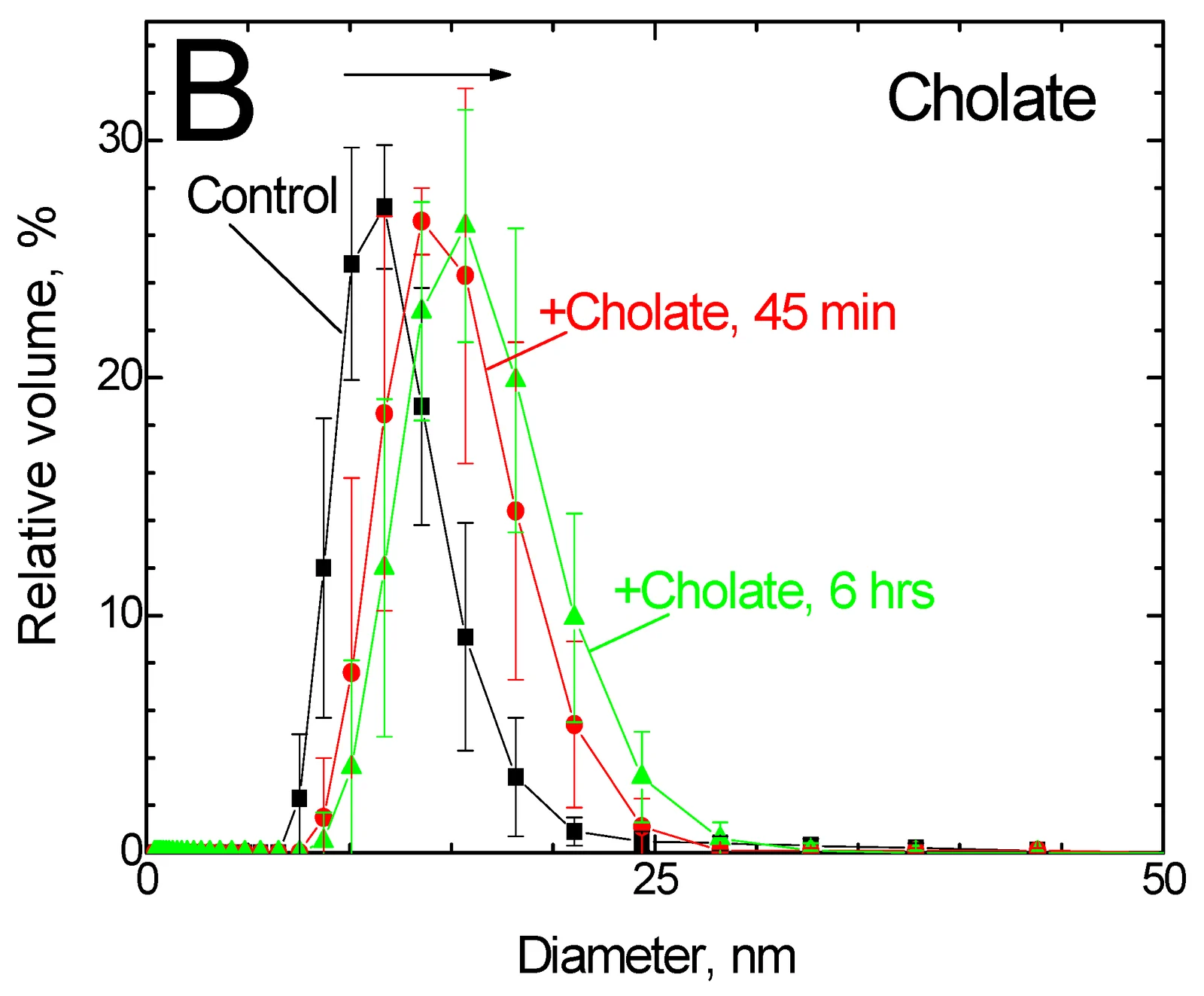
Change in micelle size in the presence of agents affecting the quaternary structure of CcO. Panels (**A**,**B**) show the results of representative experiments. Each experiment was repeated three times using three independent enzyme preparations. (**A**) Effect of Triton X-100. CcO concentration is 5 µM. Control: 0.05% Triton X-100, 50 mM potassium phosphate buffer, pH 6.5 (black squares). Monomerizing conditions: 5% Triton X-100, 100 mM CHES, pH 9.5 (red circles). (**B**) Cholate effect. Concentration of CcO is 2.8 µM (“Cholate-free” preparation, see Methods). Control: 50 mM potassium phosphate buffer, pH 7.4, 0.05% DM (black squares). Dimerizing conditions: 4 mM cholate was added to the experimental medium; recordings were made 45 min (red circles) and 6 h (green triangles) after addition.

The size distribution of CcO micelles was further studied in the presence of specific detergents that alter the quaternary structure of the protein. The classic method for monomerizing mitochondrial CcO is treatment with high Triton X-100 under alkaline conditions. Figure 3A shows the distribution of micelles in a solution containing Triton X-100 and CcO. The red curve obtained under monomerization conditions (5% = 77 mM Triton X-100, pH 9.5) has a single narrow maximum at 7.5 nm. As a control, a sample was tested in the presence of a low concentration of Triton X-100 (0.7 mM, which only slightly exceeds CMC) in a slightly acidic medium. The distribution in the control shows a maximum at 11 nm, as well as a second, very wide one, centered around 30 nm. Notably, although the distribution pattern in the control sample changed significantly upon varying CcO concentration, the shape of the curve under monomerization conditions was always reproducible, showing a single narrow maximum at 7.5 nm (see Supplementary Materials Figure S3). The control demonstrating the dependence of micelle size distribution on pH *per se* is given in Figure S4. Alkaline conditions cause a shift in the distribution curve to lower values, with the differences compared to acidic conditions leveling out as enzyme concentration decreases and detergent concentration increases.

Another modifier of mitochondrial CcO quaternary structure is cholate. It is known that a multiple excess of cholate over non-ionic detergents induces reversible dimerization of the enzyme [22]. The data in Figure 3B demonstrate the change in the size of CcO-containing micelles after the addition of cholate in a 4:1 molar excess relative to DM. It is important to note that in this experiment an additionally purified “Cholate-free” CcO preparation was used which contained, according to our estimates, no more than 2 µM of this substance (see Methods). The figure shows the dynamics of changes in the distribution curve after adding cholate to the measuring cuvette: after 45 min the maximum shifts to 14–15 nm, and after 6 h to 16–17 nm. If DM concentration in the experimental medium exceeded that of cholate, no shift in the maximum occurred (see Supplementary Materials Figure S5A). As opposed to the mitochondrial enzyme, in the case of cytochrome oxidase *aa*_3_ from *R. sphaeroides,* the addition of cholate in a 4-fold excess relative to DM did not lead to any shift in the distribution maximum (Figure S5B). Remarkably, the micelle size distribution on the “Cholate-free” CcO did not demonstrate dependence on the protein concentration. In the range 1–9 µM, the curve has a single maximum near 10 nm (Figure S5C). As an additional control, we examined the micelle size distribution over the same concentration range using the “Cholate-rich” CcO preparation containing 1% (23 mM) cholate (see Methods). At CcO concentrations above 1 μM, a single broad maximum is observed near 18 nm. (Figure S5D). 

#### 2.1.3. Size Distribution of CcO in Amphipol Preparations 

Next, we attempted to fix the quaternary state of CcO by converting the protein into a complex with amphipol (see Methods). The resulting preparations of the presumably monomeric and dimeric forms (AmM and AmD, respectively) were completely water-soluble, had a normal optical absorption spectrum, and had high cytochrome oxidase activity (see Supplementary Materials Figure S6 and Table S1, respectively). 

**Figure 4:**
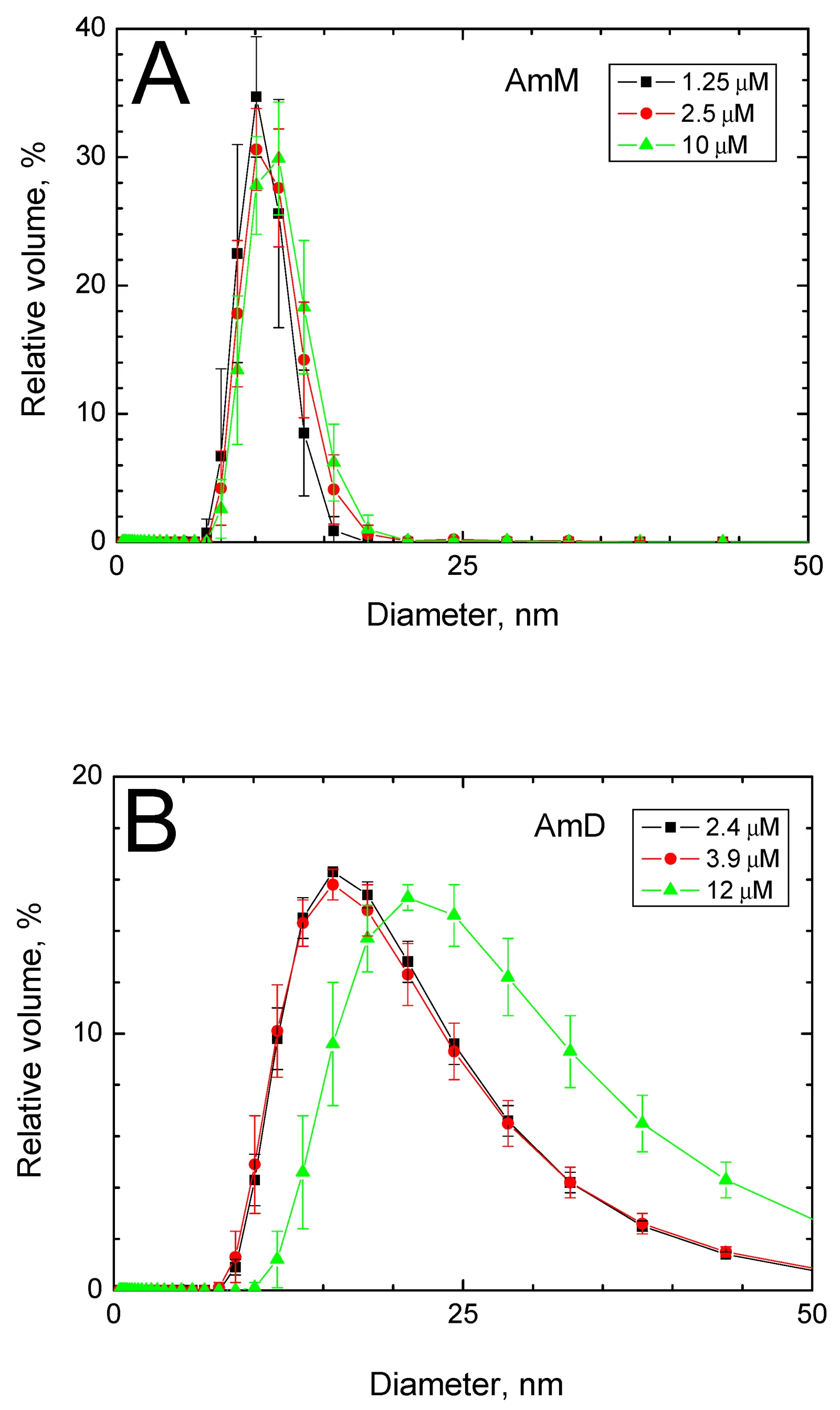
Particle size distribution in CcO samples complexed with amphipol. Experimental medium: 50 mM HEPES/NaOH, pH 7.5. The assay was conducted the day after sample preparation. Panels (**A**,**B**) show the results of representative experiments. Each measurement was triplicated. (**A**) Monomeric CcO with amphipol (AmM). CcO concentrations: 1.25 μM (black squares), 2.5 μM (red circles), 10 μM (green triangles). The experiment was repeated with two independent AmM preparations. (**B**) Dimeric CcO with amphipol (AmD). CcO concentrations: 2.4 μM (black squares), 3.9 μM (red circles), 12 μM (green triangles). The experiment was repeated with different replicates of the AmD preparation.

The results of the DLS assay on AmM and AmD samples are presented in Figure 4.

In the case of AmM (panel A), over the entire range of concentrations studied (1–10 µM CcO), the distribution of micelles has a single maximum at 9 nm, which is fairly narrow and symmetrical, indicating the homogeneity of the sample. In the case of AmD (panel B), the distribution is characterized by an asymmetric maximum at 15 nm with an extended flank on the side of larger diameters. When CcO increases to 12 µM, the maximum shifts to 20 nm (green curve).

During long-term storage of the frozen preparations, the DLS characteristics of AmM are generally preserved. In the case of AmD, the maximum shifts further to 24–25 nm and broadens on both flanks (Supplementary Materials Figure S7A). 

### 2.2. Molecular Mass of CcO as Determined by Thin-Layer Gel-Filtration Chromatography (TGCP)

**Figure 5:**
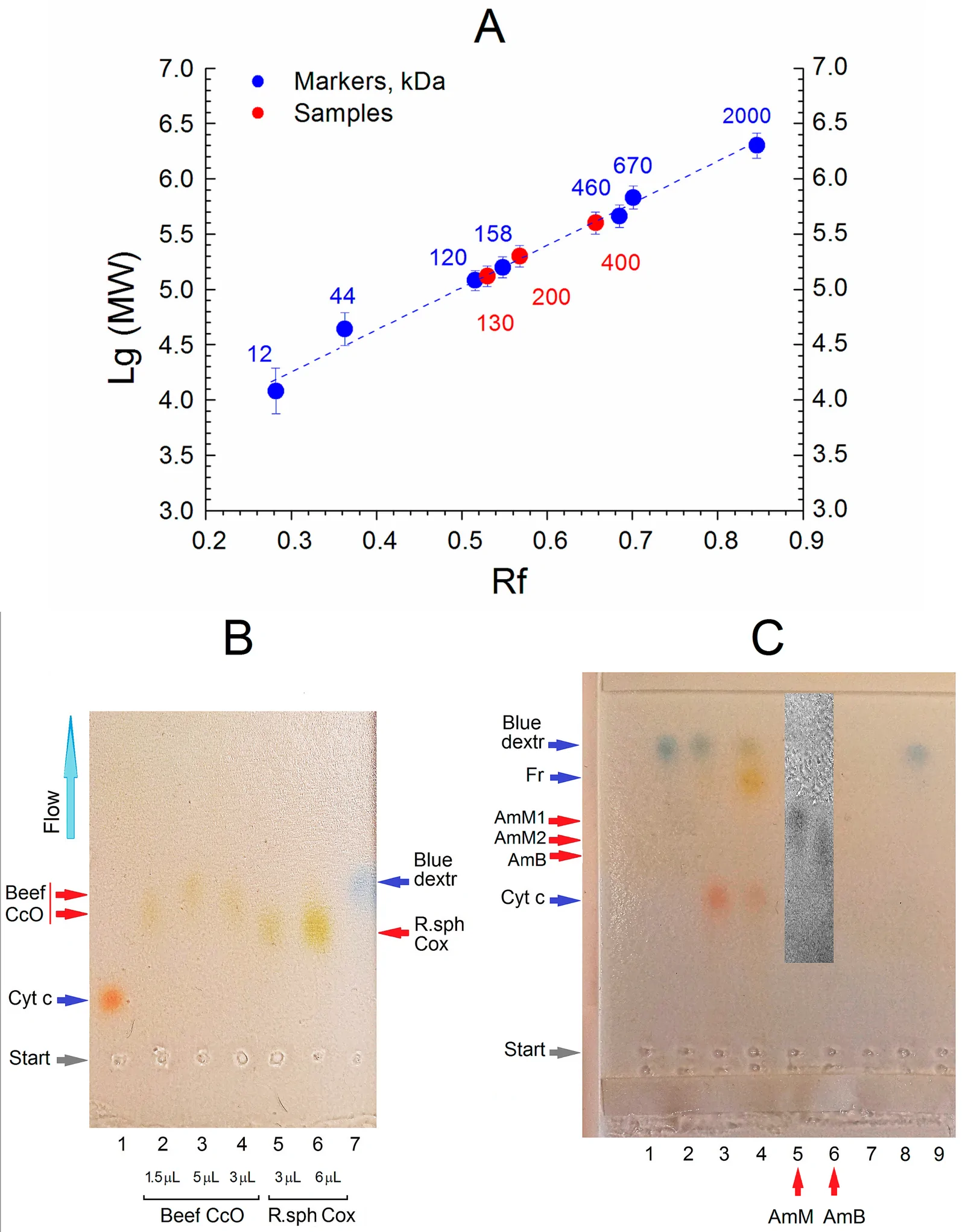
Determination of the molecular mass of CcO by thin-layer gel-filtration of proteins (TGCP). (**A**) A calibration curve was constructed using the following standards: horse heart cytochrome *c*, bovine liver catalase, horse spleen ferritin, blue dextran (MW ca. 2,000,000; Sigma, Virginia Beach, VA, USA), chicken ovalbumin, bovine γ-globulin, and bovine thyroglobulin (Bio-Rad, Hercules, CA, USA). For calculations, the 120–2000 kDa range of the calibration curve was used. Aliquots of mitochondrial CcO and the *aa*_3_-type cytochrome oxidase from *R. sphaeroides* were applied in volumes of 1–8 µL from 100 and 115 µM stock solutions, respectively. The eluent was 50 mM potassium phosphate buffer (pH 7.4) containing 0.05% DM. Colored circles denote standards (blue) and oxidase samples (red). The measurements were performed in triplicate using three independent enzyme suspensions. (**B**) Separation of mitochondrial cytochrome oxidase (CcO; 30, 100, and 60 µg of protein per lane 2–4, respectively) and bacterial cytochrome oxidase (Cox; 45 and 90 µg of protein per lanes 5 and 6, respectively) 4 h after the start of elution. Conditions were as described for panel (**A**). (**C**) Separation of amphipol complexes of mitochondrial (AmM, 67 µg of protein/lane) and bacterial (AmB, 80 µg of protein/lane) cytochrome oxidases 7 h after the start of elution. The eluent was 50 mM HEPES/NaOH (pH 7.5). Molecular-mass markers are seen in the panel as colored spots: cytochrome *c* (red), ferritin (orange), and blue dextran (blue). Cytochrome oxidase samples were detected with ninhydrin reagent on a paper replica of the gel following chromatography, as described in Methods. Colored arrows indicate the direction of elution (“Flow”; light-blue, to the left of panels (**B**,**C**)), the start of elution (gray), standards (blue), and samples (red).

In order to estimate the molecular mass of the CcO preparation obtained in the present study, TGCP was used. A set of naturally colored and a set of FITC-labeled proteins with molecular masses in the range of tens to hundreds of kDa suitable for gel-filtration analyses (see Methods), additionally supplemented with blue dextran (MW ~2000 kDa), were used as standards to construct a calibration curve (Figure 5A).

Based on the calibration curve and the Rf observed in the TGCP experiments in the presence of detergent, cytochrome oxidase from *R. sphaeroides* used in our study has an MW of approximately 130 kDa, which is in good agreement with published data. At the same time, an apparent MW of the mitochondrial CcO was determined as either 200 kDa (the approximate mass of the monomeric form) or 400 kDa (the approximate mass of the dimer), depending on the amount of protein loaded onto the gel. 

This tendency can be seen in the chromatogram (Figure 5B). The mitochondrial sample was loaded into lanes 2, 3, and 4 in various volumes of 1.5, 5, and 3 µL, respectively. During the elution, the sample in lane 3 migrated ahead of the samples in lanes 2 and 4, in which migration occurred at a slower and approximately the same rate. The mobility of the sample in lane 3 corresponds to an MW of ca. 400 kDa, whereas in lanes 2 and 4, it corresponds to ca. 200 kDa. Thus, the mitochondrial enzyme exhibits a dependence of the migration rate in the gel on the local sample concentration. We assume that this effect may be related to the process of reversible CcO dimerization during the experiment. Notably, no such effect was observed for the bacterial cytochrome oxidase used as a control alongside the mitochondrial CcO under the same conditions: compare the positions of the spots in lanes 5 and 6, which were loaded with single- and double-volume samples of *R. sphaeroides* cytochrome oxidase, respectively (Figure 5B). Upon elution, the samples in both lanes moved in parallel at a rate corresponding to an MW of 130 kDa. 

Mitochondrial and bacterial cytochrome oxidases complexed with amphipol were also analyzed using TGCP, but in this case non-detergent conditions were used (Figure 5C). It is important to note that a relatively old AmM preparation (stored at −74 °C for two months, with two freeze–thaw cycles) was investigated by TGCP. As can be seen, during elution, the mitochondrial CcO sample (AmM) resolved into two distinct spots, designated AmM1 and AmM2. One of these spots, AmM1, migrated substantially faster during elution than the cytochrome oxidase from *R. sphaeroides* (AmB). The other spot, AmM2, exhibited an intermediate migration rate, eluting between the more mobile AmM1 and the most slowly migrating AmB. Such distribution of the samples according to their mobility during TGCP suggests that AmM1 has the highest molecular mass, followed by AmM2 and then AmB, which has the lowest molecular mass among the three. In this case, the molecular masses of the samples were estimated at approximately 398, 190, and 130 kDa, respectively, which is quite close to the values determined for these cytochrome oxidases in DM micelles.

### 2.3. Native Blue Polyacrylamide Gel Electrophoresis (NB-PAGE) of CcO Complexed with Amphipol

**Figure 6:**
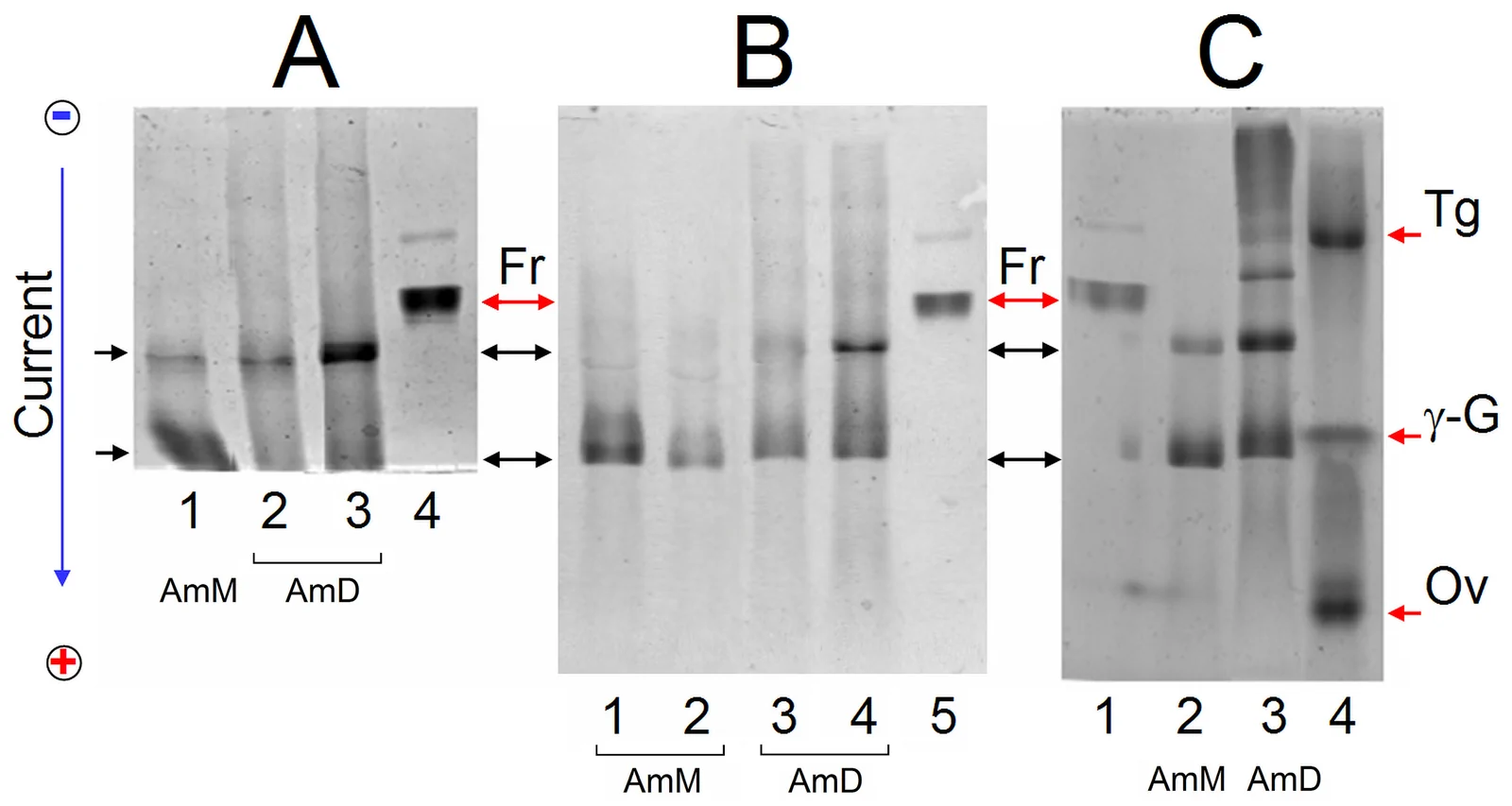
Native blue polyacrylamide gel electrophoresis (NB-PAGE) of CcO samples complexed with amphipol. Horse spleen ferritin (Fr, MW = 460 kDa) and a standard set of proteins for gel filtration: bovine thyroglobulin (Tg, MW = 670 kDa), bovine γ-globulin (γg, MW = 158 kDa), and chicken ovalbumin (Ov, MW = 44 kDa were used as markers. The positions of markers (red arrows) and protein bands of CcO samples in complex with amphipol (black arrows) are shown at the edges of the photos. The blue arrow to the left of panels (**A**–**C**) indicates the direction of electric current. (**A**) AmM (lane 1) and AmD (lanes 2 and 3) a day after preparation (frozen once in liquid nitrogen, thereafter stored at −74 °C). Lane 3: the protein concentration is twice as high as in lane 2. Lane 4: ferritin. (**B**) AmM (lanes 1 and 2) and AmD (lanes 3 and 4) ten days after preparation (see the storage information above). Lanes 1 and 4: twice the protein concentration compared to lanes 2 and 3, respectively. Lane 5: ferritin. (**C**) AmM (lane 2) and AmD (lane 3) two months after preparation, subjected to two freeze–thaw cycles. Lane 1: ferritin; lane 4: protein standard set.

Protein mobility was investigated in AmM and AmD preparations subjected to gel electrophoresis under non-denaturing conditions. The results revealed sequential changes in the sample composition over storage periods ranging from one day to two months (Figure 6A–C). The DLS data obtained for the samples shown in panels A and C (Figure 6) can be seen in Figure 4 and Figure S7A, respectively.

Two bands were observed in both samples, AmM and AmD. The less mobile band was located near the ferritin marker (lanes 1–3, panel A; lanes 1–4, panel B; lanes 2 and 3, panel C), while the more mobile band migrated to a position near the γ-globulin marker area (lane 1, panel A; lanes 1–4, panel B; lanes 2 and 3, panel C). A day after preparation, the more mobile component markedly predominated in AmM (lane 1, panel A), whereas AmD was represented almost exclusively by the less mobile component (lanes 2 and 3, panel A). After ten days of storage, the AmM composition remained unchanged (lanes 1 and 2, panel B), whereas a small amount of a more mobile component appeared in AmD (lanes 3 and 4, panel B). After two months of storage with two freeze–thaw cycles, a less mobile component was still present in AmM, and its contribution slightly increased (lane 2, panel C). At the same time, the contribution of the more mobile component increased in AmD and, furthermore, new bands with positions around the thyroglobulin marker appeared in this sample (lane 3, panel C). The latter indicated the presence of high-molecular-weight components in the AmD sample, presumably resulting from aggregation. Despite these changes over the two-month storage period, the less mobile component, localized closer to the ferritin marker, remained predominant in AmD preparation. 

Since the AmM and AmD preparations presumably contain CcO monomers and dimers, respectively, it is reasonable to assume that the more mobile band visualized in the region of the γ-globulin marker corresponds to the monomeric form of the enzyme, whereas the less mobile band, located next to the ferritin marker, corresponds to the dimeric form. Based on the data presented above, the amphipol complex of the dimer is less stable during long-term storage compared to the monomer, being particularly prone to aggregation.

### 2.4. AmM preparation as Visualized by Cryogenic Electron Microscopy (Cryo-EM)

Single-particle Cryo-EM analysis was performed to elucidate the structural organization of the studied AmM complex. To this end, a relatively fresh AmM preparation (stored at −74 °C for five days) was used. Analysis of the Cryo-EM micrographs and subsequent 2D classification confirmed that the purified sample predominantly exists in a monomeric state (Figure S8). Notably, the amphipol belt displays a width of around 4 nm and a thickness of 1.5 nm, consistent with structural findings from other studies utilizing this specific polymer [47]. Further single-particle refinement yielded a consensus map with a global resolution of 3.2 Å. Local resolution is heterogeneously distributed, reaching up to 3.2 Å within the rigid core (central region) of the complex (Figure S9), while dropping in peripheral areas due to structural flexibility. 

**Figure 7:**
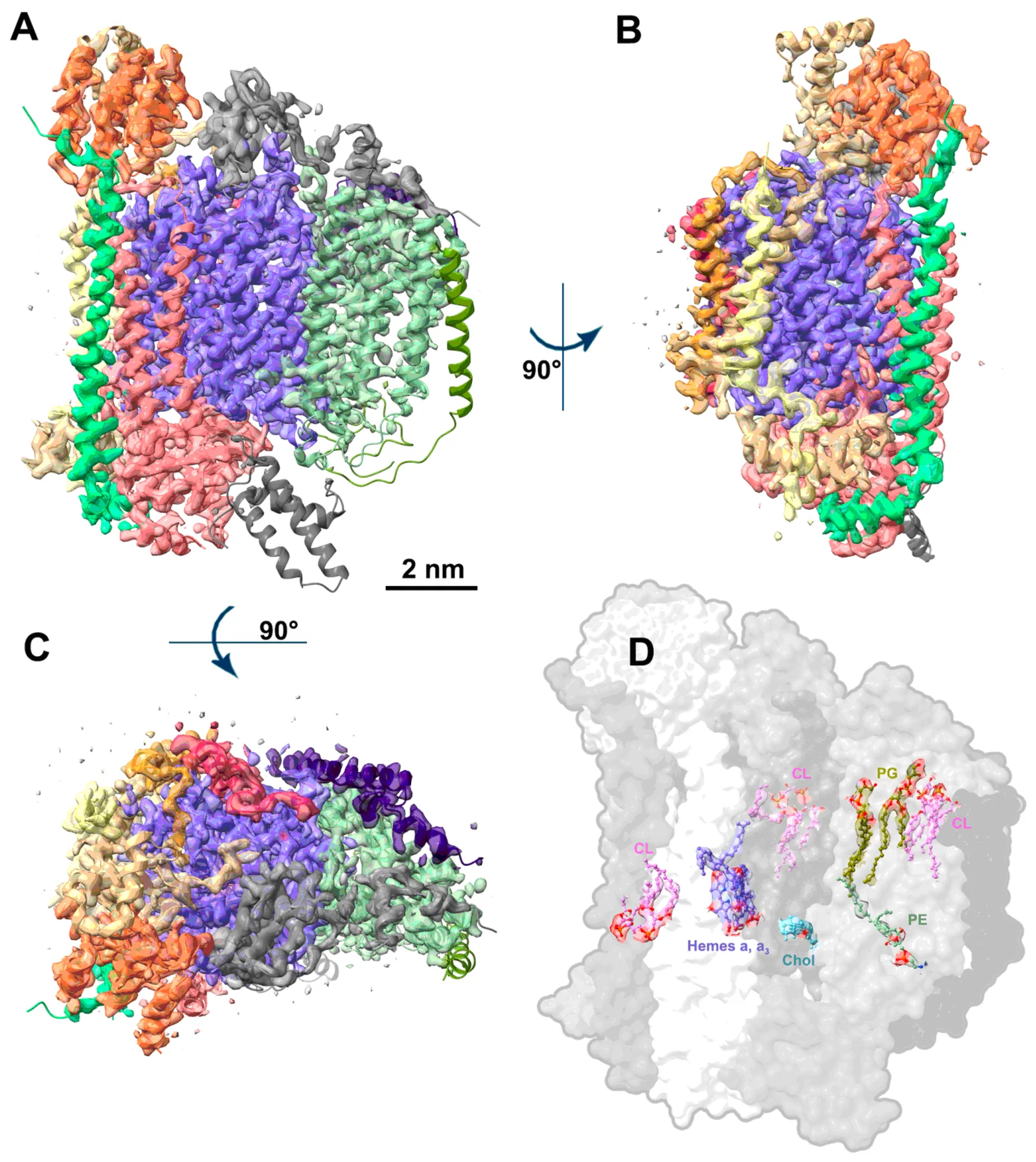
Cryo-Electron microscopy (Cryo-EM) structure of CcO complexed with amphipol. (**A**) Overview of the Cryo-EM density map of cytochrome c oxidase determined at a global resolution of 3.2 Å. The map is displayed at a fixed contour threshold optimized to exclude the surrounding polymer belt. The high-resolution atomic structure (PDB: 6JY3) is rigid-body fitted into the density and represented as α-helices. (**B**,**C**) Orthogonal views of the complex, showing the Cryo-EM density and fitted model rotated by 90° around the vertical and horizontal axes, respectively. (**D**) View of the electron densities corresponding to the non-protein components of the complex. The atomic models from PDB: 6JY3 are overlaid as a ball-and-stick model, showing alignment within the most rigid, least mobile regions. CcO subunits are colored as folows: subunit 1, lilac (panels **A**–**C**); subunit 2, pink (panels **A**,**B**); subunit 3, pale green (panels **A**,**C**); subunit 4 isoform 1, tan (panels **B**,**C**); subunit 5A, orange (panels **A**–**C**); subunit 5B, dark gray (panels **A**,**C**); subunit 6A2, forest green (right in panels **A**,**C**); subunit 6B1, gray (bottom in panels **A**,**B**); subunit 6C, lime green (panels **A**,**B**); subunit 7A1, dark lilac (panel **C**); subunit 7B, light-yellow (panels **A**–**C**); subunit 7C, pink (top in panel **C**); subunit 8B, honey-yellow (top in panel **C**). Key cofactors and lipids are labeled and colored: hemes *a* and *a_3_* (lilac), cholate (Chol, cyan), cardiolipin (CL, pink), phosphatidylethanolamine (PE, pale green), and phosphatidylglycerol (PG, olive).

The Cryo-EM density map shows excellent agreement with the α-helices of the reference crystal structure (PDB: 6JY3) across almost the entire complex (Figure 7). However, a notable discrepancy was observed for small subunits 6A and 6B, where the density is significantly blurred. Due to their high intrinsic flexibility and conformational mobility, these subunits suffered from severe local resolution degradation during the single-particle Cryo-EM averaging process. 

Despite the extraction and reconstitution of the enzyme in amphipols, several tightly bound non-protein components were preserved within our sample, mirroring the structural integrity observed in previous crystallography studies [42]. The high-quality Cryo-EM density allows for unambiguous identification of these essential elements (Figure 7D). Beyond the structural hemes, which serve as the core catalytic cofactors of the enzyme, distinct densities were resolved for various structural lipids, including cardiolipin (CL), phosphatidylglycerol (PG), and phosphatidylethanolamine (PE). Interestingly, a density corresponding to a cholate molecule bound at the protein’s outer periphery was also clearly visible. Notably, the binding sites for all identified lipids and cofactors perfectly aligned with previously reported positions, highlighting the structural preservation of the complex in its native-like functional state.

## 3. Discussion

DLS analysis of CcO in detergent solution reproducibly revealed two discrete maxima in the particle-size distribution under various conditions: at 7–10 nm and within the 16–24 nm range. The first maximum is relatively narrow and more symmetrical compared to the second. A conventional sphere with a diameter of 7–10 nm is comparable to the linear dimensions of monomeric CcO (Figure 1). However, this coincidence does not prove that the maximum at 7–10 nm characterizes a monomeric preparation. Quantitative estimates based on DLS data are sensitive not only to size but also to other particle characteristics, most notably to their shape, which in the case of CcO is clearly not spherical. Nevertheless, our results provide a strong indication that the maximum of 7–10 nm actually corresponds to the monomeric state of the enzyme. This indication comes from the following combination of circumstances. 

First, there is a significant body of indirect yet mutually consistent data demonstrating the appearance of a peak in the 7–10 nm range under conditions that promote monomerization. This includes factors such as a decrease in CcO concentration (Figure 2B), an increase in the concentration of non-ionic detergents in the medium (Figure S2), and alkaline conditions (Figure S4). A convincing control is provided by bacterial oxidase, which, as opposed to mitochondrial CcO, retains a monomeric state even at high concentrations [6]. In accordance with this observation, solubilized *aa*_3_-type cytochrome oxidase from *R. sphaeroides* exhibits a distribution with a single maximum near 10 nm, the position of which does not depend on the enzyme concentration (Figure 2C). 

Then, it is even more telling that under classical conditions of mitochondrial CcO monomerization (treatment with 5% TX-100 in alkaline medium [32]), the entire enzyme population is converted to a form characterized by a distribution with a maximum at 7–10 nm, regardless of the previous state (Figure 3A and Figure S3).

Finally, the AmM sample obtained using the protocol for preparing the monomeric form of the enzyme in complex with amphipol [29,42] is characterized in DLS studies by a single peak at 9–10 nm, regardless of protein concentration. Cryo-EM data unequivocally confirm that this AmM preparation contains CcO monomers in complex with amphipol (Figure 7) exclusively.

The origin of the maximum at ≥16 nm is more difficult to interpret unambiguously. Its exact position varies under different experimental conditions. Addition of cholate to the “Cholate-free” CcO induces a peak shift from 10 nm to 16–17 nm (Figure 3B). A similar position of the maximum is observed for the particle size distribution of the AmD sample obtained at a high cholate concentration (Figure 4B, black and red curves). Since excess cholate relative to DM is known to induce reversible dimerization of the mitochondrial enzyme, it is reasonable to assume that the observed micelles, approximately 16–17 nm in size, correspond to CcO dimers.

Data obtained from two additional independent methods support the hypothesis that the shift in the distribution maximum from 7 to 10 nm to 16 nm and above is associated with the reversible dimerization of CcO. First, TGCP chromatography revealed in the solubilized CcO samples two components with MWs of 200 kDa and 400 kDa, which correspond well to the monomeric and dimeric forms of the enzyme [48] (Figure 5A). Moreover, it was established that the migration rate of the component during chromatography depends on the amount of sample applied to the gel (i.e., on the local CcO concentration). An increase in concentration caused a discrete shift in the apparent MW from 200 to 400 kDa (Figure 5B). This observation is in agreement with DLS data, according to which an increase in CcO concentration in the solution is accompanied by a discrete shift in the particle-size distribution maximum to the right. An MW value close to 200 kDa was obtained by the same method for the AmM sample, which, according to Cryo-EM data, contains the monomeric enzyme.

Second, non-denaturing gel electrophoresis revealed two protein components present at reciprocal relative abundances in the AmM and AmD preparations. The more mobile component predominated markedly in AmM, whereas the less mobile component was predominant in AmD (Figure 6). Comparing these data with the DLS results for the same samples (Figure 4), it can be inferred that the more mobile component corresponds to the peak at 7–10 nm, while the less mobile one corresponds to the peak at 16–17 nm. Since Cryo-EM data indicate that fresh AmM sample consists solely of the monomeric form, it is evident that the component with higher electrophoretic mobility corresponds to the monomer. The second, less mobile protein component predominating in AmD most likely corresponds to a dimer. It is noteworthy that prolonged storage and repeated freeze–thaw cycles resulted in the formation of high-molecular-weight aggregates in the AmD preparation, detectable by both NB-PAGE and DLS (Figure 6C and Figure S7A). The AmM preparation is clearly much more resistant to aggregation. However, it contains the AmD form as a minor impurity, the contribution of which increases slightly during storage (Figure 6).

A number of factors apparently promote the shift in the second maximum detected by DLS further to the right: to 21–22 nm (the AmD sample at a high concentration, see the green curve in Figure 4B), 24–25 nm (AmD after long-term storage with repeated freezing/thawing, the red curve in Figure S7A) and 24–26 nm (CcO in a 0.05% DM at high protein concentrations, Figure 2B). Moreover, high enzyme concentration and acidic environment cause the appearance of additional minor maxima at values of 30 nm and above (see purple curve in Figure 2B and black curve in Figure 3A). We suppose that the shift in the second maximum toward larger sizes is attributable to aggregation. Probably, the dimeric form of CcO exhibits a higher affinity for binding amphipathic compounds from solution (such as detergent or amphipol molecules, as well as CcO in various states) as compared to the monomeric form. Therefore, it may be hypothesized that the aggregation process is triggered by the preliminary dimerization of the enzyme.

The shape of the second maximum also varies depending on the conditions. The asymmetry of the particles themselves can contribute to the asymmetry of the particle size distribution measured by DLS. From this perspective, one would expect the CcO dimer (less symmetrical than the monomer, see Figure 1) to exhibit a less symmetrical size distribution. This is precisely what is observed (Figure 4, cf. A vs. B). The second obvious reason for asymmetry may be the aggregation of particles, both among themselves and with the smaller ones. Accordingly, in long-stored AmD samples containing protein aggregates, DLS detects a significant broadening of the distribution toward higher values (Figure S7A; compare with Figure 4B).

To summarize, it can be stated that in some of our experiments (under the conditions of cholate-induced dimerization), the DLS technique seems to reveal the true dimeric form of CcO. The absence of a cholate-dependent distribution shift in the case of bacterial oxidase indicates that the action of cholate is specific and the observed increase in particle size is not due to random aggregation. In other cases, the method apparently registers the simultaneous presence of the dimeric form and its aggregates, which is reflected by a broadening of the right flank of the distribution, a shift in the maximum toward higher values, and an increase in the area under the curve.

The possibility of fixing the quaternary structure of CcO with amphipol seems to be very promising. The amphipol complex of the monomeric enzyme appears to be particularly stable, retaining its properties during long-term storage. Fresh preparations of the dimeric enzyme with amphipol appear to be stable enough to conduct experiments. This opens the possibility of studying CcO in definitely monomeric and dimeric forms.

A series of our recent studies was devoted to the regulatory site of mitochondrial CcO that binds amphipathic ligands [34,49,50,51]. This site is presumed to be the Bile Acid–Binding Site (BABS), first described in the X-ray crystal structure of the dimer by the Ferguson-Miller group [52,53,54]. According to structural data, canonical BABS can only exist in the dimeric form of the enzyme. Excitingly, for the monomeric form of mitochondrial CcO, another regulatory site that binds steroids and is located at a distance of about 20 Å from BABS was described in the published study of Di Trani *et al.* [55]. The presence of functionally similar but spatially separated regulatory sites inherent in different quaternary forms of CcO appears to be an interesting case of crossing regulatory pathways [56]. Further study of this phenomenon requires enzyme preparations with a fixed quaternary structure that does not change during experiments. In this regard, monomeric and dimeric CcO complexed with amphipol may prove particularly useful.

In general, our results indicate that DLS is a suitable and convenient technique for real-time monitoring of changes in the quaternary structure of solubilized CcO.

## 4. Materials and Methods

### 4.1. Chemicals

High-purity reagents of foreign and domestic production were used, including: pH buffers HEPES, Tris and CHES, potassium and sodium phosphates, ammonium sulphate (Amresco, Solon, OH, USA); cholic acid, Triton X-100, sodium dithionite, cytochrome *c* (type III from equine heart), TMPD (N,N,N’,N’-tetramethyl-*p*-phenylenediamine), L-ascorbic acid, blue dextran, Coomassie G-250 (Sigma, St. Louis, MO, USA); chicken ovalbumin, bovine γ-globulin, bovine thyroglobulin, Bio-Beads particles (Bio-Rad, Hercules, CA, USA); n-dodecyl β-D-maltoside (Anatrace, Maumee, OH, USA); Amphipol 8–35 (Thermo-Fisher, Waltham, MA, USA); Sephadex G-200 superfine (Pharmacia, Uppsala, Sweden); sucrose (high purity grade H-1807, Trilab, Moscow, Russia). 

### 4.2. Isolation of Cytochrome Oxidases and Sample Preparations

Isolation of mitochondrial CcO from bovine heart mitochondria was carried out according to Fowler et al. [20] with minor modifications as described in [21]. After separation of respiratory complexes I and III, the precipitated CcO was solubilized with cholate (1 mg/mg of protein) and then precipitated with increasing concentrations of ammonium sulfate. The precipitate was solubilized with 0.05% DM in 50 mM HEPES/Tris buffer (pH 8.0). The resulting CcO preparation was estimated to contain 1–2 mM cholate impurity. 

Typically, preparations had an enzyme concentration of 100–200 µM (determined spectrophotometrically) and a protein concentration of 25–50 mg/mL (determined by the standard Biuret method). The purity of the samples was confirmed by the high relative heme content (7.5–8 nmol of heme *a* per mg of protein). Cryo-EM analysis revealed the complete subunit composition of the monomeric enzyme in our preparation, with the exception of the NDUFA4 subunit (which is typically absent with the given isolation procedure). 

A “Cholate-free” CcO preparation was further purified from cholate by a 1000-fold dilution. For this purpose, the sample was concentrated using Amicon Ultra cut-off 100 kDa mini-filters (Millipore, Billerica, MA, USA) to a minimum volume and diluted 10-fold with a medium containing 50 mM HEPES/Tris (pH 8.0) and 0.05% DM. The procedure was repeated three times.

A “Cholate-rich” CcO preparation was obtained using the method described above, including precipitation of the enzyme with ammonium sulfate. The precipitate was solubilized in cholate (final concentration, 2%) and subsequently purified by ultracentrifugation through a linear 5–20% sucrose gradient prepared in 0.5 M potassium phosphate buffer (pH 7.4) [57]. The final preparation contained cholate at a concentration of approximately 1% (23 mM).

Monomeric CcO complexed with amphipol was prepared according to the method described for the terminal oxidase *bo*_3_ from *Escherichia coli* [29]. The isolated CcO was diluted to a final concentration of 7.5 µM in a medium containing 50 mM HEPES/Tris (pH 8.5), 50 mM KCl, and 0.05% DM. Then, amphipol A8–35 was added to the protein at a threefold excess by weight. The sample was gently shaken for 4 h at 7 °C. Subsequently, activated Bio-Bead particles were added up to 20 mg/mL, and incubation was continued for 12 h. The resulting sample was concentrated 5 times using Amicon Ultra cut-off 100 kDa mini-filters. 

Dimeric CcO complexed with amphipol was prepared according to the previously described method [58] with the following modifications. Initially, CcO was converted into cholate micelles. For this purpose, 5–15 mg of protein isolated using the standard procedure was dialyzed against 250 mL of a medium containing 100 mM sodium phosphate (pH 7.5) and 1% sodium cholate for 15 h at 7 °C. Thereafter, amphipol A8–35 was added to the protein at a threefold excess by weight, and the preparation was gently mixed for 30 min at 0 °C. Subsequently, the detergent was removed using β-cyclodextrin by dialyzing the sample for 2 h against 250 mL of a medium containing 100 mM HEPES/NaOH (pH 7.8) and 1% β-cyclodextrin. After 1 h, the buffer was replaced with a fresh one. Following dialysis, the preparation was centrifuged through a 1.1–1.35 M sucrose gradient in a medium containing 40 mM HEPES/NaOH (pH 7.8) for 20 h at 100,000× *g*.

Cytochrome oxidase *aa*_3_ from *R. sphaeroides* was isolated from membranes of the wild-type strain in which subunit I of the enzyme had been modified by the addition of a hexahistidine tag (His-tag) to its C-terminus. Isolation was performed on a Ni-NTA agarose column as described earlier [59].

The *R. sphaeroides* cytochrome oxidase–amphipol complex was prepared using a procedure analogous to that employed for the monomeric mitochondrial CcOamphipol complex (see above). The final sample was concentrated using Amicon Ultra cut-off 30 kDa mini-filters.

All samples prepared as described above were stored at −74 °C after preliminary freezing in liquid nitrogen.

The optical spectra of the CcO preparations examined in this study are presented in the Supplementary Materials Figure S6. Absorption spectra were recorded at room temperature with a Cary 300 Bio spectrophotometer (Varian, Palo Alto, CA, USA) in a semi-micro 1 mL optical glass cuvette with a window width of 4 mm, blackened walls and 10 mm light pathway (Hellma, Müllheim, Germany), in a double-beam mode, at a speed of 2 nm/s and a slit width of 2 nm. Difference spectra were obtained by subtracting the spectrum of the sample oxidized by atmospheric oxygen from the spectrum of the sample reduced with dry sodium dithionite. 

Data on the enzymatic activity of the samples are presented in Table S1. Cytochrome oxidase activity was estimated by oxygen consumption rates using a covered Clark-type electrode of an Oxytherm instrument (Hansatech, King’s Lynn, Norfolk, UK), in a thermostated chamber maintained at 25 °C under continuous stirring. As the oxidation substrate, 5 mM ascorbate, 0.1 mM TMPD and 5 μM cytochrome *c* were used. The sample was added to achieve a final cytochrome oxidase concentration of 30–50 nM. The activity of the solubilized enzyme was measured in a medium containing 50 mM Hepes, 50 mM Tris (pH 7.5), 0.1 mM EDTA, and 0.05% DM. Enzyme activity in the amphipol complex was measured in a detergent-free medium containing 50 mM Hepes/NaOH (pH 7.5).

### 4.3. DLS Measurements and Analyses

DLS was registered with a commercial Zetasizer Nano ZS instrument (Malvern Instruments, Malvern, Worcestershire, UK) at 25 °C. A helium-neon laser operating at 633 nm served as a light source. The sample was placed in a cuvette (optical path 10 mm, final volume 100 µL) and incubated at the specified temperature for 1–3 min, after which data collection began. One experiment included 10 measurements, each consisting of ten 25 s recordings. The results were processed using the Dispersion Technology version 5.10 Software supplied with the instrument. The error of the results was estimated as the standard deviation of the averaged data. 

The DLS experiments presented in this study were generally repeated 3 times using independent enzyme preparations (see figure captions for details).

The key data acquisition parameters for the representative samples are shown in Figure S10.

### 4.4. Thin-Layer Gel-Filtration Chromatography of Protein Samples

TGCP was carried out according to the method proposed by Wieland et al. [60] and later summarized by Devenyi and Gergely [61], with our modifications. Sephadex G-200 superfine (Pharmacia, Uppsala, Sweden) was used as a carrier. Swelling of the dry gel was carried out for the first 2 h at 50 °C and then left for 24 h at room temperature. To swell the gel, the same medium was used in which the experiment was subsequently conducted (2 g of dry gel per 100 mL of liquid medium). Flat glass panels measuring 11 × 11 cm and 0.3 cm thick, with 0.2 cm high and 0.4 cm wide borders along the entire right and left sides, were used as the chromatography chamber. The working side of the chambers, together with the borders, was covered with a single matte opaque thin white plastic film. Before pouring into the chamber, excess liquid was drained from the glass container with the swollen gel; the gel suspension was stirred with a glass rod; and then evenly distributed over the entire working surface of the chamber. The glass rod was then applied to the protruding side borders of the chamber on the left and right, and the gel was quickly rolled along the entire length of the chamber. The chamber with the filled gel was then left wrapped in Saran Wrap for 1–2 days to dry at room temperature. Wells for applying samples were made using a sampler, pointwise taking out 6 µL from each well. To ensure the flow of eluent through the gel, paper wicks (3MM-type paper) were placed at the inlet and outlet of the chamber, where there are no side borders. The distal end of the wick at the outlet should extend 2–3 cm beyond the chamber edge and extend below the chamber level into the receiving reservoir so that the edge of the wick touches the thin layer of eluent placed there. After loading the samples into the wells, the chamber tilt was set to 15° from the upper inlet edge to the lower outlet edge to ensure downward elution. Elution was performed at 20 °C, with the eluent supplied uniformly to the inlet wick at a flow rate of 0.08 mL/min. The naturally colored proteins (see Figure 5) and a standard set of marker proteins for gel filtration (Bio-Rad, USA) fluorescently labeled with fluorescein isothiocyanate (FITC) (Lumiprobe, Moscow, Russia) were used as markers. When it was necessary to detect proteins after separation, filter paper was placed over the corresponding tracks in the chromatographic chamber, onto which protein samples were transferred, and the resulting replica was stained with ninhydrin reagent using standard methods described by Devenyi and Gergely [61].

### 4.5. NB-PAGE

Disk electrophoresis was performed under non-denaturing conditions using the standard method with a concentrating 5%-PAG and a separating 6–15%-gradient-PAG at a current of 20–30 mA at 4 °C for 5–6 h. Tris-glycine buffer with the addition of 0.5% Coomassie G-250 dye served as a cathode buffer. Subsequent staining of the gels with Coomassie G-250 was carried out using the standard method. 

### 4.6. Cryo-Electron Microscopy and Image Processing

Cryo-EM sample preparation and data collection were performed as follows. Quantifoil R1.2/1.3 Cu grids were glow-discharged for 20 s at 20 mA (0.26 mbar) using a PELCO easiGlow system. A 3 µL-aliquot of the AmM suspension (1 mg/mL) was applied to the grids, blotted for 3 s at 100% humidity and 4 °C, and plunge-frozen in liquid ethane using a Vitrobot Mark IV (Thermo Fisher Scientific, Waltham, MA, USA). Cryo-EM data were collected on a Titan Krios 60–300 transmission electron microscope (Thermo Fisher Scientific, USA) equipped with a field emission gun (X-FEG), a spherical-aberration corrector (CEOS GmbH, Heidelberg, Germany), a post-column BioQuantum energy filter (Gatan, Pleasanton, CA, USA), and a K3 direct electron detector (Gatan, Pleasanton, CA, USA) operating in counting mode. SerialEM 4.0 was used for data acquisition at 300 kV with a nominal magnification of 81,000×, corresponding to a calibrated pixel size of 0.874 Å at the specimen level. An energy-selecting slit of 20 eV was used. A total dose of 52 e^−^/Å^2^ was fractionated into 70 frames over a 3.5 s exposure time. A total of 1011 movies were collected in a nominal defocus range from −0.7 to −1.5 µm with a step of 0.1 µm.

For Cryo-EM image processing, drift correction, contrast transfer function (CTF) estimation, particle picking, and particle extraction were performed using Warp 1.9. All subsequent processing was carried out in CryoSPARC 4.6. A set of 20,739 particles, extracted with a box size of 256 pixels, was subjected to 2D classification. The resulting class averages showed no evidence of dimeric CcO species, confirming the homogeneity of the monomeric preparation. These particles were then subjected to *ab initio* reconstruction with two classes at a maximum resolution of 6 Å, which yielded a major class of 7428 particles that produced a reconstruction consistent with the expected monomeric structure. The remaining particles, which did not converge to a well-defined density, were excluded from further analysis. The selected 7428 particles were refined using an initial round of Non-Uniform Refinement, followed by CTF Refinement and a second round of Non-Uniform Refinement. The final Cryo-EM map of the monomeric CcO–amphipol complex (AmM) was reconstructed at an overall resolution of 3.19 Å, as estimated by the Fourier shell correlation (FSC) criterion at 0.143. The Cryo-EM map of AmM has been deposited in the Electron Microscopy Data Bank (EMDB) with accession code EMD-83586.

### 4.7. Data Presentation

DLS and TGCP data were further processed for final presentation using Microcal Origin, versions 7 and 9 (https://www.originlab.com/). PyMOL Molecular Graphics System, Version 1.2r1 Schrodinger, LLC was used to visualize the 3D structures. USFC ChimeraX was used for the figure showing Cryo-EM densities [62].

## 5. Conclusions

Depending on the experimental conditions, two main patterns of CcO size distribution in micelles are detected by the DLS techniques. A rather symmetrical distribution with a narrow maximum at 7.5–10 nm is promoted by conditions that stabilize the monomeric state of the enzyme. In the corresponding CcO sample, a single protein component with a molecular mass of approximately 200 kDa is identified, which still predominates upon conversion of the enzyme into a complex with amphipol (AmM preparation). AmM is characterized by a concentration-independent size distribution with a maximum in the 7.5–10 nm range, similar to solubilized bacterial cytochrome oxidase. Cryo-EM imaging confirms that the AmM preparation contains exclusively monomeric CcO. Thus, the maximum at 7.5–10 nm most likely corresponds to the monomeric state of the enzyme.

The second distribution pattern has a rather wide and asymmetrical peak at 16 nm or above. It is observed under conditions that promote reversible dimerization of CcO. In the corresponding samples, a single or dominant protein component with a molecular mass ca. 400 kDa is identified. For the enzyme in the presence of excess cholate, as well as for the state obtained by cholate treatment and stabilized in a complex with amphipol (the AmD preparation), the distribution has a maximum at 16–17 nm. This value can probably be attributed to the dimeric form. The shift toward even higher values is driven by factors promoting non-stoichiometric particle aggregation, such as an acidic environment, prolonged storage, or repeated freeze–thaw cycles. This aggregation process is apparently triggered by the initial formation of dimers.

Thus, the DLS method can register changes associated with the reversible dimerization of CcO in solution and identify individual quaternary states of the enzyme.

## Data Availability

The Cryo-EM map of the monomeric CcO–amphipol complex (AmM) has been deposited in the Electron Microscopy Data Bank (EMDB) under accession code EMD-83586. Other data obtained in this study are contained in the main text of the article and in the Supplementary Materials. Further inquiries can be directed to the authors.

## Supplementary Materials

The following supporting information can be downloaded at: https://www.mdpi.com/article/10.3390/ijms27198744/s1.

## Abbreviations

The following abbreviations are used in this manuscript:

AmB	Cytochrome oxidase *aa*_3_ from *R. sphaeroides* in complex with amphipol
AmD	Dimeric CcO in complex with amphipol
AmM	Monomeric CcO in complex with amphipol
BABS	Bile acid binding site
CcO	Cytochrome *c* oxidase
CMC	Critical micelle concentration
Cryo-EM	Cryogenic electron microscopy
DLS	Dynamic light scattering
DM	n-Dodecyl β-D-maltoside
MW	Molecular mass
NB-PAGE	Native blue polyacrylamide gel electrophoresis
Rf	Retardation factor in chromatography
TGCP	Thin-layer gel-filtration chromatography of proteins
TN	Turnover number of the enzyme

## References

[1] Rich P.R. (2017). Mitochondrial cytochrome *c* oxidase: Catalysis, coupling and controversies. Biochem. Soc. Trans..

[2] Wikström M., Sharma V. (2018). Proton pumping by cytochrome *c* oxidase—A 40 year anniversary. Biochim. Biophys. Acta (Bioenerg.).

[3] Siletsky S.A., Borisov V.B. (2021). Proton pumping and non-pumping terminal respiratory oxidases: Active sites intermediates of these molecular machines and their derivatives. Int. J. Mol. Sci..

[4] Hendler R.W., Pardhasaradhi K., Reynafarje B., Ludwig B. (1991). Comparison of energy-transducing capabilities of the two-and three-subunit cytochromes *aa_3_* from *Paracoccus denitrificans* and the 13-subunit beef heart enzyme. Biophys. J..

[5] Sousa F.L., Alves R.J., Ribeiro M.A., Pereira-Leal J.B., Teixeira M., Pereira M.M. (2012). The superfamily of heme–copper oxygen reductases: Types and evolutionary considerations. Biochim. Biophys. Acta (Bioenerg.).

[6] Svensson-Ek M., Abramson J., Larsson G., Törnroth S., Brzezinski P., Iwata S. (2002). The X-ray crystal structures of wild-type and EQ (I-286) mutant cytochrome c oxidases from *Rhodobacter sphaeroides*. J. Mol. Biol..

[7] Qin L., Hiser C., Mulichak A., Garavito R.M., Ferguson-Miller S. (2006). Identification of conserved lipid/detergent-binding sites in a high-resolution structure of the membrane protein cytochrome *c* oxidase. Proc. Natl. Acad. Sci. USA.

[8] Love B., Chan S.H., Stotz E. (1970). Molecular weight of two states of cytochrome *c* oxidase. J. Biol. Chem..

[9] Takemori S., Sekuzu I., Okunuki K. (1961). Studies on cytochrome a VII. Physico-chemical properties of purified cytochrome *a*. Biochim. Biophys. Acta.

[10] Tzagoloff A., Yang P.C., Wharton D.C., Rieske J.S. (1965). Studies on the electron-transfer system LX. Molecular weights of some components of the electron transfer chain in beef-heart mitochondria. Biochim. Biophys. Acta (Nucleic Acids Protein Synth.).

[11] Hakvoort T.B., Moolenaar K., Lankvelt A.H., Sinjorgo K.M., Dekker H.L., Muijsers A.O. (1987). Separation, stability and kinetics of monomeric and dimeric bovine heart cytochrome *c* oxidase. Biochim. Biophys. Acta (Bioenerg.).

[12] Okunaki K., Sekuzu I., Yonetani T., Takemori S. (1958). Studies on cytochrome A. I. Extraction, purification and some properties of cytochrome A. J. Biochem..

[13] Yonetani T., Takemori S., Sekuzu I., Okunuki K. (1958). Activation of cytochrome oxidase by synthetic detergents. Nature.

[14] Horie S., Morrison M., Bright J. (1963). Cytochrome *c* Oxidase Components: I. Purification and properties. J. Biol. Chem..

[15] Van Buuren K.J.H., Van Gelder B.F. (1974). Biochemical and biophysical studies on cytochrome *c* oxidase. XIII. Effect of cholate on the enzymic activity. Biochim. Biophys. Acta (Bioenerg.).

[16] Yu C., Yu L., King T.E. (1975). Studies on cytochrome oxidase. Interactions of the cytochrome oxidase protein with phospholipids and cytochrome *c*. J. Biol. Chem..

[17] Yonetani T. (1961). Studies on Cytochrome Oxidase: III. Improved Preparation and Some Properties. J. Biol. Chem..

[18] Soulimane T., Buse G. (1995). Integral cytochrome-*c* oxidase. Preparation and progress towards a three-dimensional crystallization. Eur. J. Biochem..

[19] Griffiths D.E., Wharton D.C. (1961). Studies of the electron transport system: XXXV. Purification and properties of cytochrome oxidase. J. Biol. Chem..

[20] Fowler L.R., Richardson S.H., Hatefi Y. (1962). A rapid method for the preparation of highly purified cytochrome oxidase. Biochim. Biophys. Acta.

[21] Vygodina T.V., Kirichenko A., Konstantinov A.A. (2014). Cation binding site of cytochrome *c* oxidase: Progress report. Biochim. Biophys. Acta.

[22] Musatov A., Robinson N.C. (2002). Cholate-induced dimerization of detergent- or phospholipid-solubilized bovine cytochrome *c* oxidase. Biochemistry.

[23] Sedlak E., Kozar T., Musatov A. (2020). The interplay among subunit composition, cardiolipin content, and aggregation state of bovine heart cytochrome *c* oxidase. Cells.

[24] Tsukihara T., Aoyama H., Yamashita E., Tomizaki T., Yamaguchi H., Shinzawa-Itoh K., Nakashima R., Yaono R., Yoshikawa S. (1996). The whole structure of the 13-subunit oxidized cytochrome *c* oxidase at 2.8 Å. Science.

[25] Lee S.J., Yamashita E., Abe T., Fukumoto Y., Tsukihara T., Shinzawa-Itoh K., Ueda H., Yoshikawa S. (2001). Intermonomer interactions in dimer of bovine heart cytochrome *c* oxidase. Acta Crystallogr. D Biol. Crystallogr..

[26] Koepke J., Olkhova E., Angerer H., Muller H., Peng G., Michel H. (2009). High resolution crystal structure of *Paracoccus denitrificans* cytochrome *c* oxidase: New insights into the active site and the proton transfer pathways. Biochim. Biophys. Acta (Bioenerg.).

[27] Lyons J.A., Aragão D., Slattery O., Pisliakov A.V., Soulimane T., Caffrey M. (2012). Structural insights into electron transfer in *caa*_3_-type cytochrome oxidase. Nature.

[28] Abramson J., Riistama S., Larsson G., Jasaitis A., Svensson-Ek M., Laakkonen L., Puustinen A., Iwata S., Wikström M. (2000). The structure of the ubiquinol oxidase from *Escherichia coli* and its ubiquinone binding site. Nat. Struct. Biol..

[29] Guo Y., Karimullina E., Emde T., Otwinowski Z., Borek D., Savchenko A. (2023). Monomer and dimer structures of cytochrome *bo_3_* ubiquinol oxidase from *Escherichia coli*. Protein Sci..

[30] Chan S.H., Love B., Stotz E. (1970). Absorption spectra and enzymatic properties of monomer and dimer states of cytochrome *c* oxidase. J. Biol. Chem..

[31] Georgevich G., Darley-Usmar V.M., Malatesta F., Capaldi R.A. (1983). Electron transfer in monomeric forms of beef and shark heart cytochrome *c* oxidase. Biochemistry.

[32] Robinson N.C., Talbert L. (1986). Triton X-100 induced dissociation of beef heart cytochrome *c* oxidase into monomers. Biochemistry.

[33] Sadoski R.C., Zaslavsky D., Gennis R.B., Durham B., Millett F. (2001). Exposure of bovine cytochrome *c* oxidase to high triton X-100 or to alkaline conditions causes a dramatic change in the rate of reduction of compound F. J. Biol. Chem..

[34] Oleynikov I.P., Azarkina N.V., Vygodina T.V., Konstantinov A.A. (2021). Mechanism of inhibition of cytochrome *c* oxidase by Triton X-100. Biochemistry.

[35] Kadenbach B. (2017). Regulation of Mammalian 13-Subunit Cytochrome *c* Oxidase and Binding of other Proteins: Role of NDUFA4. Trends Endocrinol. Metab..

[36] Ramzan R., Rhiel A., Weber P., Kadenbach B., Vogt S. (2019). Reversible dimerization of cytochrome *c* oxidase regulates mitochondrial respiration. Mitochondrion.

[37] Ramzan R., Kadenbach B., Vogt S. (2021). Multiple mechanisms regulate eukaryotic cytochrome *c* oxidase. Cells.

[38] Althoff T., Mills D.J., Popot J.L., Kuhlbrandt W. (2011). Arrangement of electron transport chain components in bovine mitochondrial supercomplex I1III2IV1. EMBO J..

[39] Genova M.L., Lenaz G. (2014). Functional role of mitochondrial respiratory supercomplexes. Biochim. Biophys. Acta (Bioenerg.).

[40] Lopez-Fabuel I., Le Douce J., Logan A., Bolaños J.P. (2016). Complex I assembly into supercomplexes determines differential mito chondrial ROS production in neurons and astrocytes. Proc. Natl. Acad. Sci. USA.

[41] Zong S., Wu M., Gu J., Liu T., Guo R., Yang M. (2018). Structure of the intact 14-subunit human cytochrome c oxidase. Cell Res..

[42] Shinzawa-Itoh K., Sugimura T., Misaki T., Tadehara Y., Yamamoto S., Hanada M., Yano N., Nakagawa T., Uene S., Yamada T. (2019). Monomeric structure of an active form of bovine cytochrome *c* oxidase. Proc. Natl. Acad. Sci. USA.

[43] Shinzawa-Itoh K., Muramoto K. (2021). Biochemical and crystallographic studies of monomeric and dimeric bovine cytochrome *c* oxidase. Biophys. Physicobiol..

[44] Popot J.L., Althoff T., Bagnard D., Banères J.L., Bazzacco P., Billon-Denis E., Catoire L.J., Champeil P., Charvolin D., Cocco M.J. (2011). Amphipols from A to Z. Annu. Rev. Biophys..

[45] Zoonens M., Popot J.L. (2014). Amphipols for each season. J. Membr. Biol..

[46] Oliver R.C., Lipfert J., Fox D.A., Lo R.H., Doniach S., Columbus L. (2013). Dependence of micelle size and shape on detergent alkyl chain length and head group. PLoS ONE.

[47] Zampieri V., Gobet A., Robert X., Falson P., Chaptal V. (2021). CryoEM reconstructions of membrane proteins solved in several amphipathic solvents, nanodisc, amphipol and detergents, yield amphipathic belts of similar sizes corresponding to a common ordered solvent layer. Biochim. Biophys. Acta.

[48] Liko I., Degiacomi M.T., Mohammed S., Yoshikawa S., Schmidt C., Robinson C.V. (2016). Dimer interface of bovine cytochrome *c* oxidase is influenced by local posttranslational modifications and lipid binding. Proc. Natl. Acad. Sci. USA.

[49] Oleynikov I.P., Azarkina N.V., Vygodina T.V., Konstantinov A.A. (2020). Interaction of cytochrome *c* oxidase with steroid hormones. Cells.

[50] Oleynikov I.P., Sudakov R.V., Azarkina N.V., Vygodina T.V. (2022). Direct Interaction of Mitochondrial Cytochrome c Oxidase with Thyroid Hormones: Evidence for Two Binding Sites. Cells.

[51] Oleynikov I.P., Sudakov R.V., Radyukhin V.A., Arutyunyan A.M., Azarkina N.V., Vygodina T.V. (2023). Interaction of Amphipathic Peptide from Influenza Virus M1 Protein with Mitochondrial Cytochrome Oxidase. Int. J. Mol. Sci..

[52] Qin L., Mills D.A., Buhrow L., Hiser C., Ferguson-Miller S. (2008). A Conserved steroid binding site in cytochrome *c* oxidase. Biochemistry.

[53] Hiser C., Buhrow L., Liu J., Kuhn L., Ferguson-Miller S. (2013). A conserved amphipathic ligand binding region influences K-path-dependent activity of cytochrome *c* oxidase. Biochemistry.

[54] Buhrow L., Hiser C., van Voorst J.R., Ferguson-Miller S., Kuhn L.A. (2013). Computational prediction and in vitro analysis of potential physiological ligands of the bile acid binding site in cytochrome *c* oxidase. Biochemistry.

[55] Di Trani J.M., Moe A., Riepl D., Saura P., Kaila V.R., Brzezinski P., Rubinstein J.L. (2022). Structural basis of mammalian complex IV inhibition by steroids. Proc. Natl. Acad. Sci. USA.

[56] Azarkina N.V., Borisov V.B., Oleynikov I.P., Sudakov R.V., Vygodina T.V. (2023). Interaction of terminal oxidases with amphipathic molecules. Int. J. Mol. Sci..

[57] Finel M., Wikstrom M. (1986). Studies on the role of the oligomeric state and subunit III of cytochrome oxidase in proton translocation. Biochim. Biophys. Acta (Bioenerg.).

[58] Shinzawa-Itoh K., Shimomura H., Yanagisawa S., Shimada S., Takahashi R., Oosaki M., Ogura T., Tsukihara T. (2016). Purification of Active Respiratory Supercomplex from Bovine Heart Mitochondria Enables Functional Studies. J. Biol. Chem..

[59] Mitchell D.M., Gennis R.B. (1995). Rapid purification of wildtype and mutant cytochrome *c* oxidase from *Rhodobacter sphaeroides* by Ni^2+^-NTA affinity chromatography. FEBS Lett..

[60] Wieland T., Lüben G., Determann H. (1962). Neue Anwendungen der Dünnschichtchromatographie [New uses of thin layer chromatography]. Experientia.

[61] Devenyi T., Gergely J. (1974). Amino Acids, Peptides and Proteins: Biochemical and Immunochemical Techniques in Protein Chemistry.

[62] Meng E.C., Goddard T.D., Pettersen E.F., Couch G.S., Pearson Z.J., Morris J.H., Ferrin T.E. (2023). UCSF ChimeraX: Tools for structure building and analysis. Protein Sci..

