# Supplementary material for: Reversible Dimerization of Cytochrome Oxidase in Detergent Solution as Detected by Dynamic Light Scattering
Source: Int J Mol Sci. 2026 Sep 30;27(19):8744. doi: 10.3390/ijms27198744 (PMC13664831; doi:10.3390/ijms27198744)
Supplement: Supplementary file 1 [file ijms-27-08744-s001.zip › ijms-4539175-supplementary.pdf]

Supplementary material for

Reversible Dimerization of Cytochrome Oxidase in Detergent Solution as Detected by Dynamic Light Scattering

Natalia V. Azarkina*, Victor N. Orlov, Maria S. Muntyan, Yury M. Chesnokov, Semen V. Nesterov, Ilya P. Oleynikov, Tatiana V. Vygodina*

* Correspondence authors: azarkina@yahoo.com (N.V.A.); vygodina@belozersky.msu.ru (T.V.V.)

Figure S1. Stability of the size distribution of CcO in DM micelles. Experimental medium: 50 mM potassium phosphate, pH 7.4, 0.05% DM, 1 μM CcO. The control black curve (squares) was obtained immediately after sample preparation. The red curve (circles) was obtained after 4.5 h of sample incubation in the experimental medium on ice. Next, 100 mM KCl was added, and the green curve (triangles) was recorded. The experiment presented was performed in duplicate with different enzyme preparations.

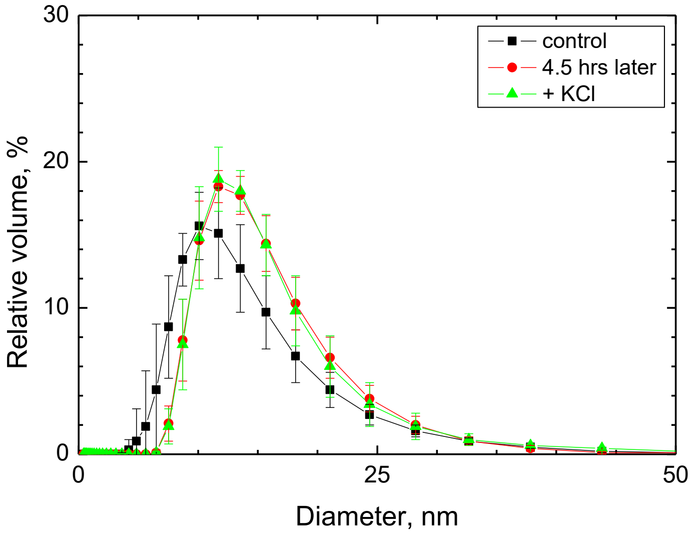

Figure S2. Size distribution of CcO in micelles depending on the concentration of protein and detergent. Experimental medium: 50 mM potassium phosphate, pH 7.4.
(**A**) 0.2% DM (4 mM). CcO concentrations: 1 μM (black squares), 10 μM (red circles), 40 μM (blue squares), 60 μM (green triangles). The blue curve was obtained after 60 min incubation of 40 μM CcO in the experimental medium at room temperature; the other curves were obtained immediately after diluting the sample to the indicated concentrations. The experiment presented was performed in triplicate with different enzyme preparations.
(**B**) 4 μM CcO. DM concentrations: 0.05% = 1 mM (black squares), 0.15% = 3 mM (red circles), 0.3% = 6 mM (green triangles). The measurements were repeated three times.

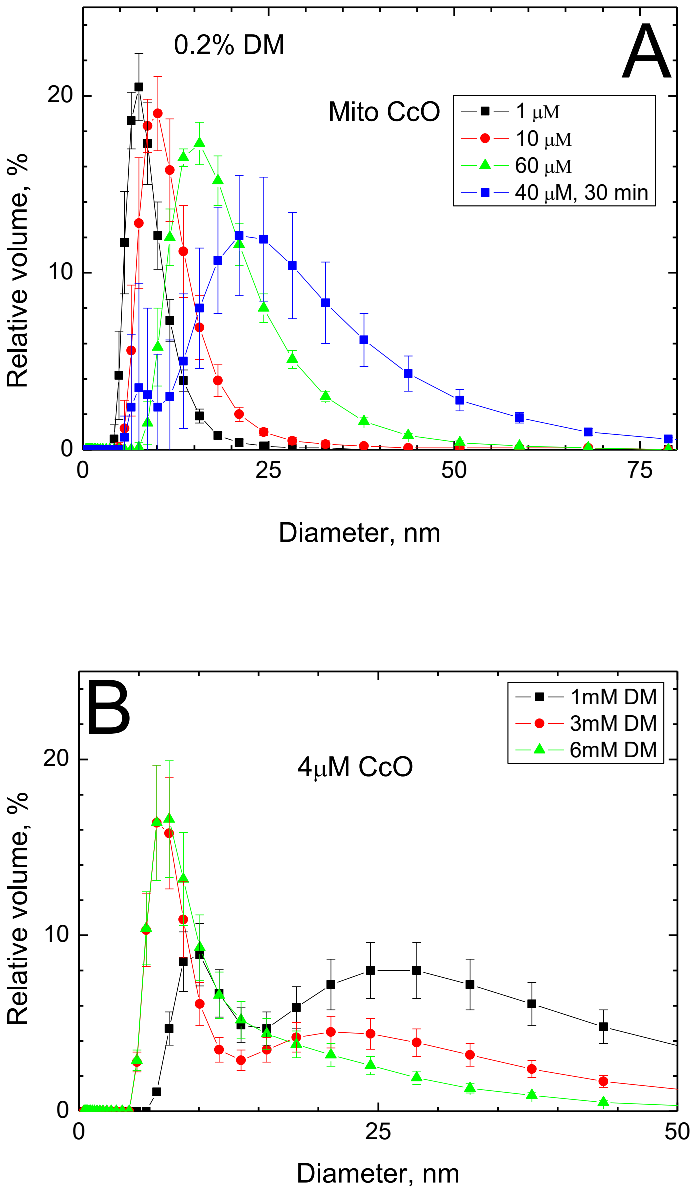

Figure S3. Monomerization of CcO by treatment with high Triton X-100 under alkaline conditions. Experimental medium: 50 mM potassium phosphate. Control: 1 μM CcO, 0.05% TX100, pH 6.6 (black squares). Monomerization conditions: 5% Triton X-100 (TX100), pH 8.6 (red circles). The experiment was triplicated with two different enzyme preparations.

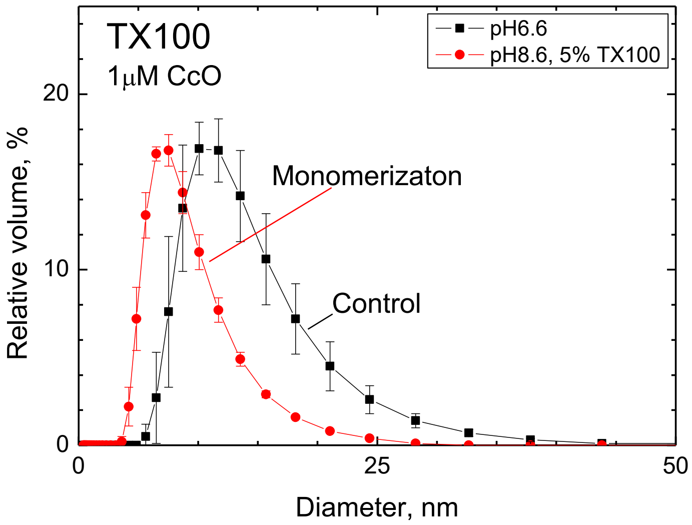

Figure S4. Size distribution of CcO in micelles at different pH. Experimental medium: 50 mM potassium phosphate. The experiments shown in panels A and B were performed on the independent replicates of the same enzyme preparation.

(**A**) 2 μM CcO, 0.05% DM, pH 6.6 (black squares) vs. pH 9.0 (red circles).

(**B**) 1 μM CcO, 0.2% DM, pH 6.5 (black squares) vs. pH 8.5 (red circles).

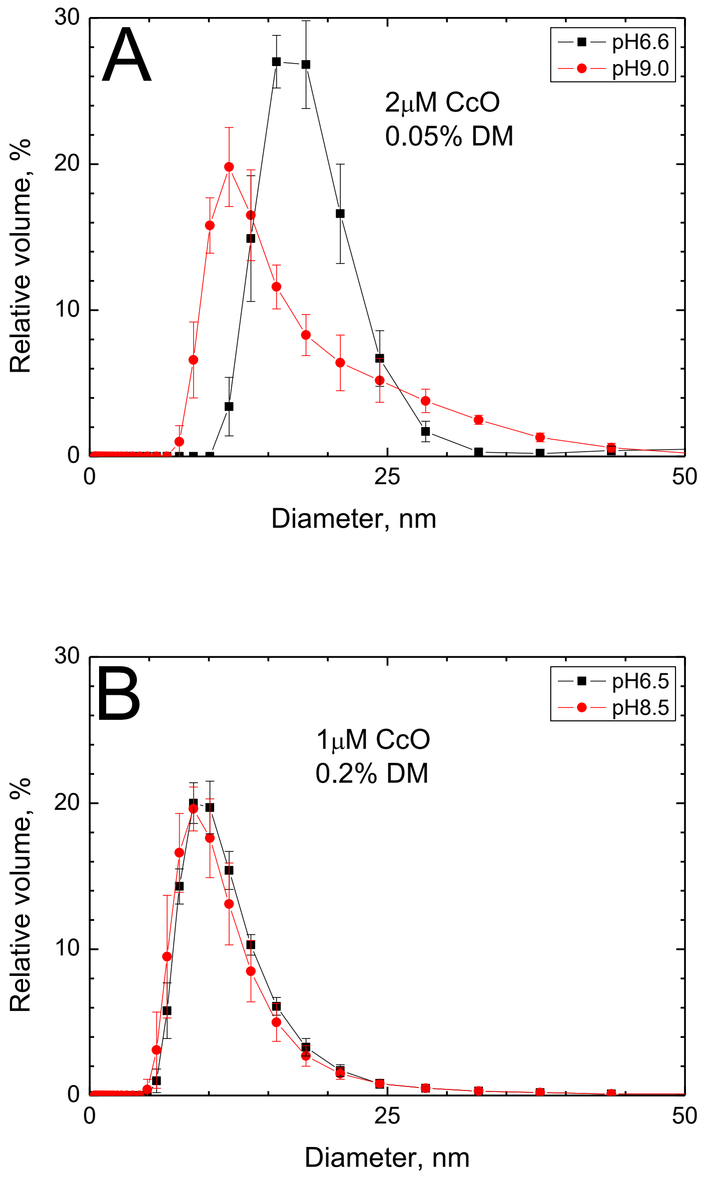

Figure S5. Effect of cholate on the size distribution of CcO in micelles. Experimental medium: 50 mM potassium phosphate, pH 7.4.

(**A**) Excess of DM relative to cholate. CcO concentration 1.1 µM. Control data (black squares) were obtained in a medium containing 0.2% DM (4 mM) and lacking cholate. Then, 2 mM cholate was added to the sample, and the red curve (circles) was recorded after 1 h. The experiment was duplicated.

(**B**) Distribution of micelles with cytochrome oxidase *aa*$_3$ from *R. sphaeroides* (4 µM) in a DM solution (0.05% = 1 mM). Control (black squares): medium does not contain cholate. Conditions of excess cholate relative to DM (red circles): 4 mM cholate was added to the same sample, data were recorded 45 min after addition. The experiment was duplicated using different enzyme preparation.

(**C**) "Cholate-free" CcO preparation (see the text) in different concentrations. The DM concentration is 0.05%. CcO concentrations are 1 µM (black squares), 2.8 µM (red circles), and 9 µM (green triangles). The measurements were duplicated.

(**D**) "Cholate-rich" CcO preparation (see the text) in different concentrations. The experimental medium contains 4 mM cholate and 0.05% DM. The symbols corresponding to different CcO concentrations are the same as in panel C. The measurements were duplicated.

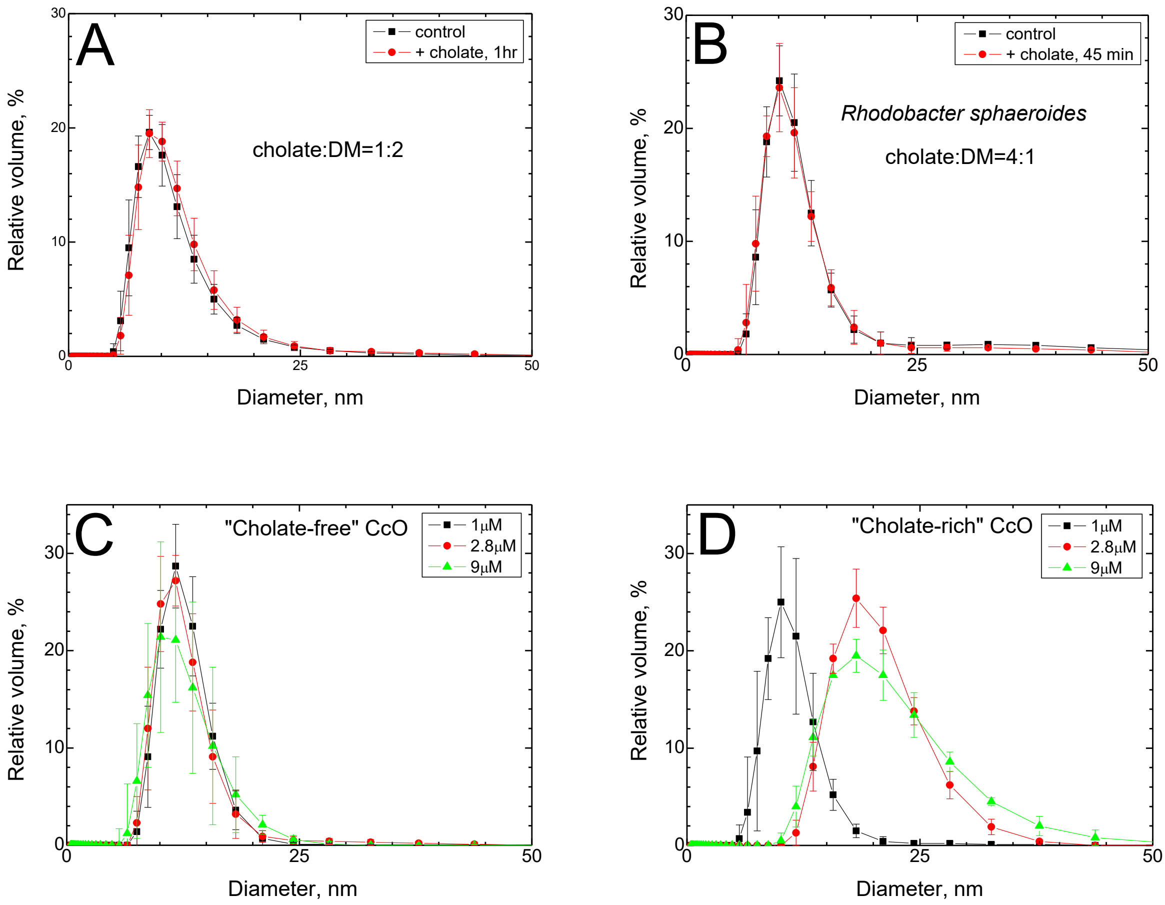

Figure S6. Optical absorption difference (reduced *minus* oxidized sample) spectra of the typical preparations investigated in this work. CcO – DM solubilized mitochondrial CcO, CcO R. sph. – DM solubilized cytochrome oxidase *aa*3 from *R. sphaeroides*, AmD – dimeric CcO in the amphipol complex, AmB – cytochrome oxidase *aa*3 from *R. sphaeroides* in the amphipol complex, AmM – monomeric CcO in the amphipol complex. The spectra are normalized to the protein concentration of the samples durng recording (0.1–0.5 mg/mL) and thus correspond to 1 mg/mL. See Methods for other conditions.

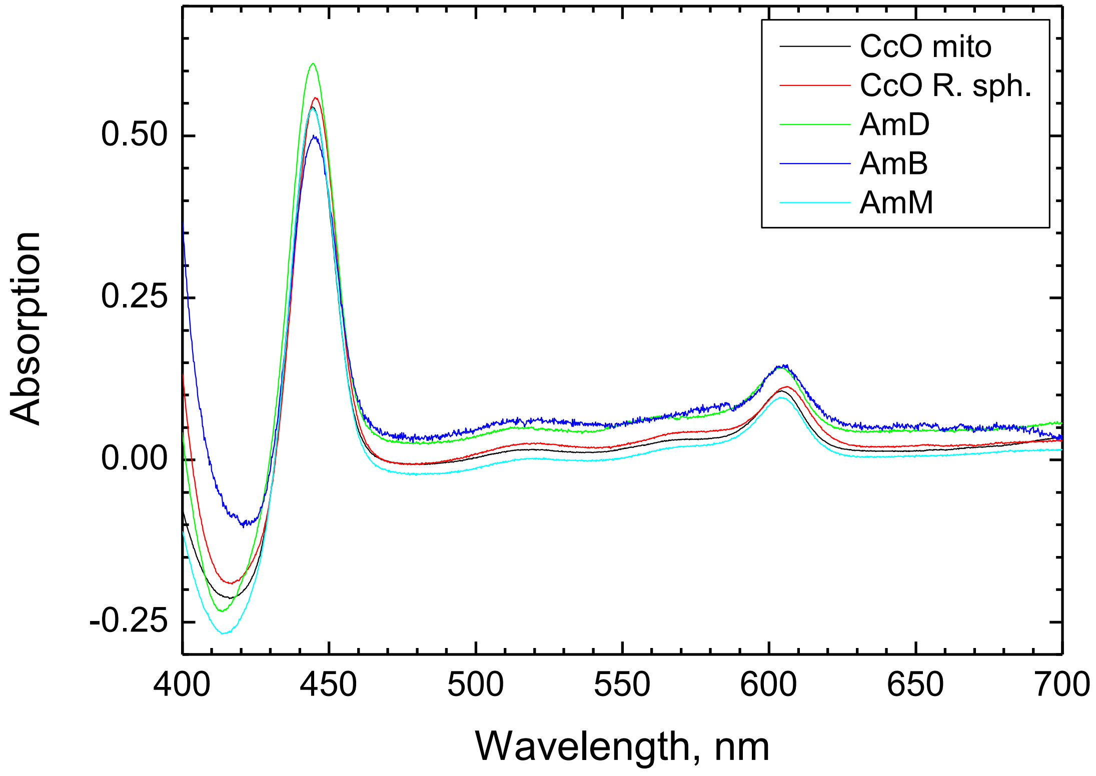

Figure S7. Particle size distribution in CcO preparations with amphipol. Experimental medium: 50 mM HEPES/NaOH, pH 7.5.
(**A**) Mitochondrial enzyme, concentration 4 μM. Data were obtained on AmM (black squares) and AmD (red circles) preparations used in native electrophoresis after 2 months of storage (Figure 6, panel C, lanes 2 and 3, respectively).
(**B**) Bacterial enzyme, 2.2 μM (black squares) and 10 μM (red circles). Data were obtained on the AmB preparation (see Figure 5C for TGCP data).

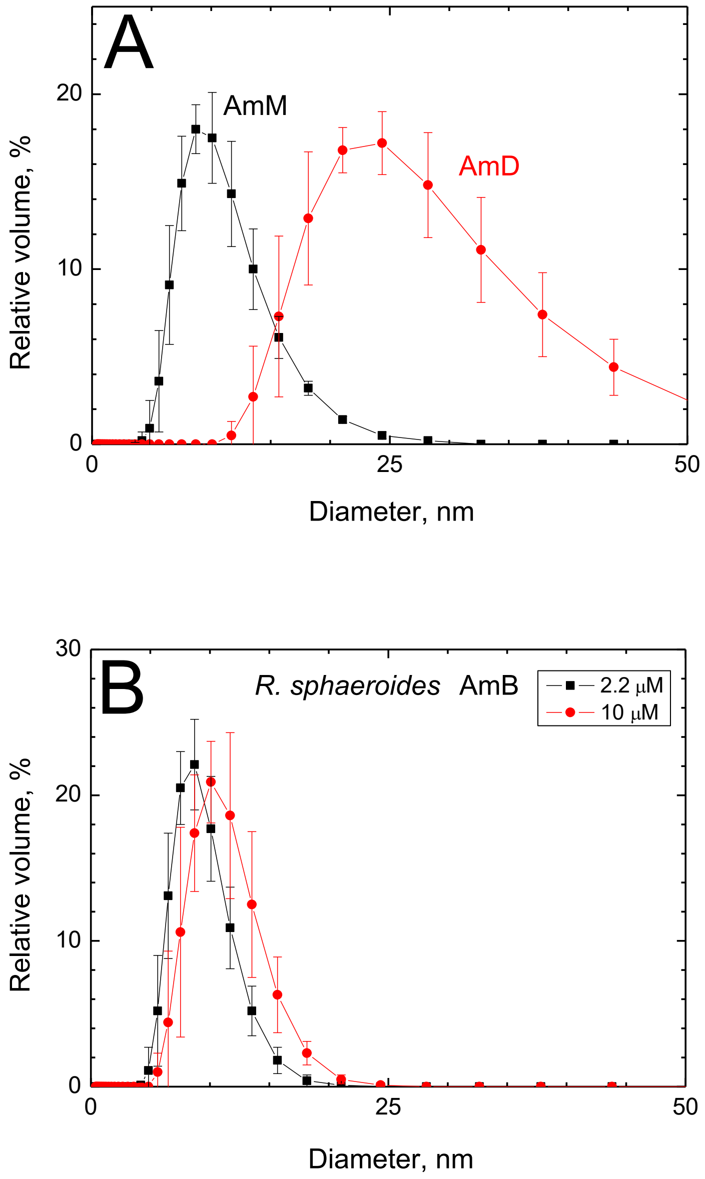

Figure S8. Cryo-EM analysis of AmM.

(**A**) Representative Cryo-EM micrograph of vitrified AmM particles. Selected particles are indicated with white arrows.

(**B**) Reference-free 2D class averages of the AmM particles. The class averages exhibit characteristic features of monomeric cytochrome *c* oxidase, with no evidence of dimeric species.

(**C**, **D**) Central slices through the final Cryo-EM map of the AmM complex, displayed in two orthogonal planes. An additional belt-shaped density, corresponding to the amphipol A8–35 shell, is clearly visible surrounding the transmembrane region of the monomer. The width of this density is measured at approximately 15–20 Å.

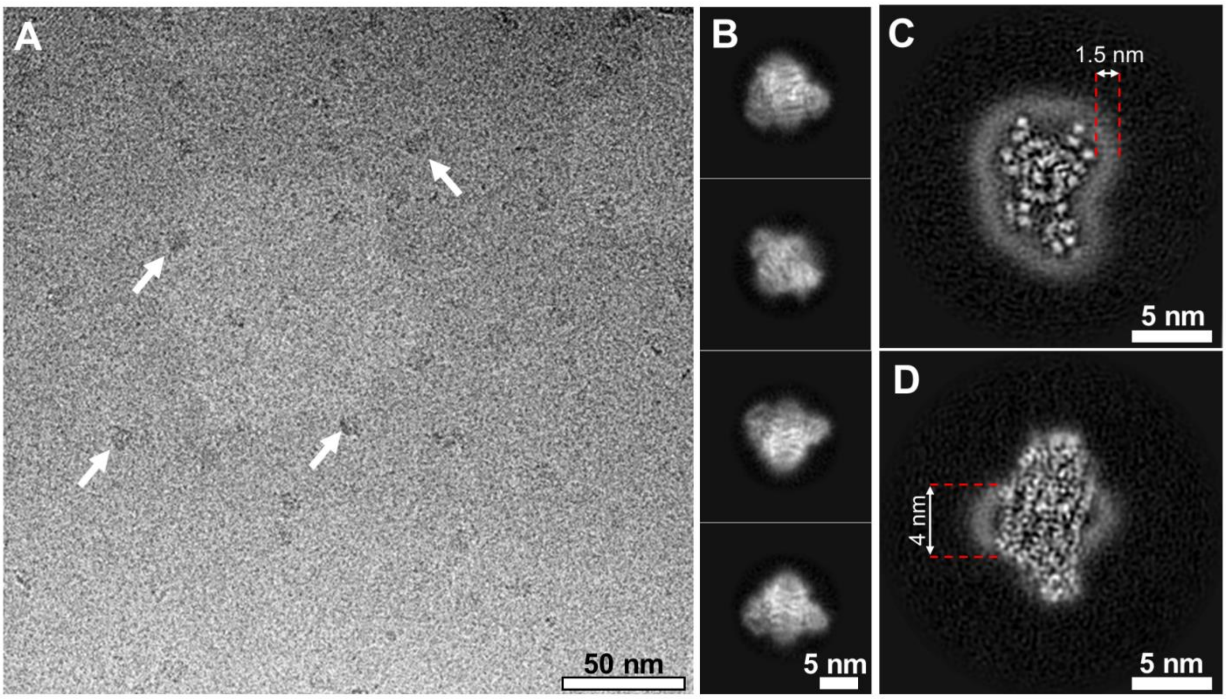

Figure S9. Cryo-EM map quality assessment for the AmM reconstruction.

(**A**) Fourier shell correlation (FSC) curve showing the overall resolution of the final map. The resolution is estimated at 3.19 Å based on the FSC = 0.143 criterion, as indicated by the horizontal solid black line on the graph.

(**B**) Angular distribution plot of the particles used in the final reconstruction. The color scale indicates the number of particles assigned to each viewing direction.

(**C**) Local resolution estimation mapped onto the Cryo-EM density of the AmM complex. The map is colored according to local resolution (from high to low, blue to red).

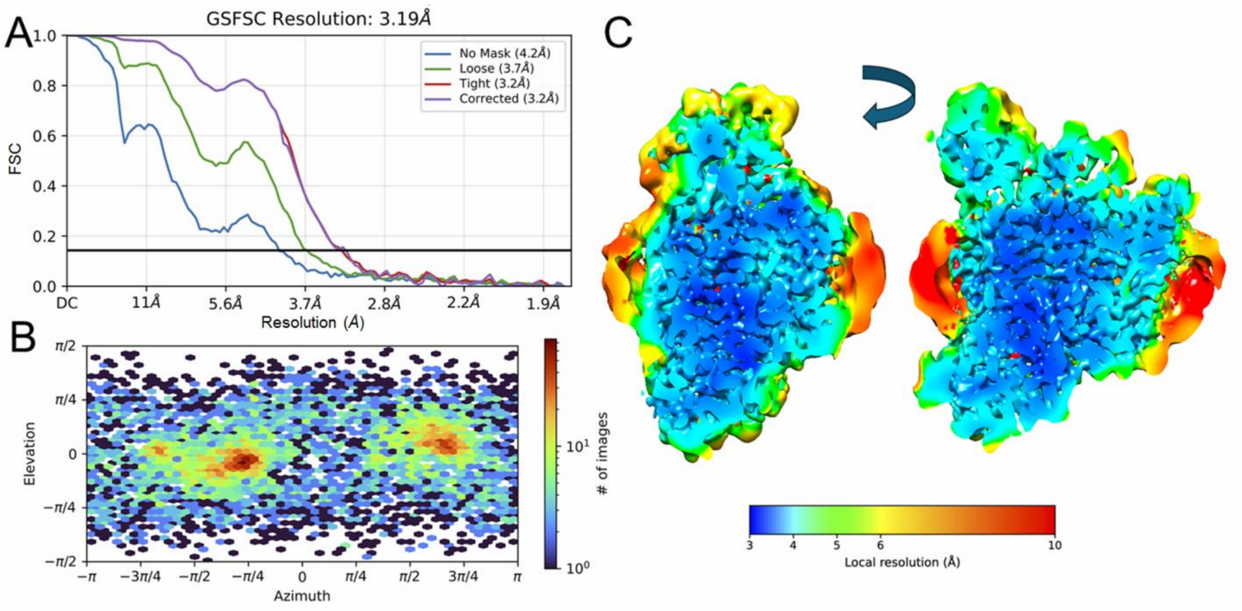

Figure S10. Example of DLS data collection on representative samples. Size distribution results and key data acquisition parameters are presented for 32 μM (sample 1) and 1.6 μM (sample 2) CcO in DM micelles. The sample 2 was produced from the sample 1 by dilution. Experimental medium: 50 mM potassium phosphate, pH 7.4, 0.05% DM.

(**A**) – Sample 1. The volume-weighted (black squares) and the intensity-weighted (red circles) distributions are shown together with the count rates and attenuation values

(**B**) – Sample 1. Z-average trends together with the polydispersity index values.

(**C**) – Sample 1. Correlogramms of the individual measurements.

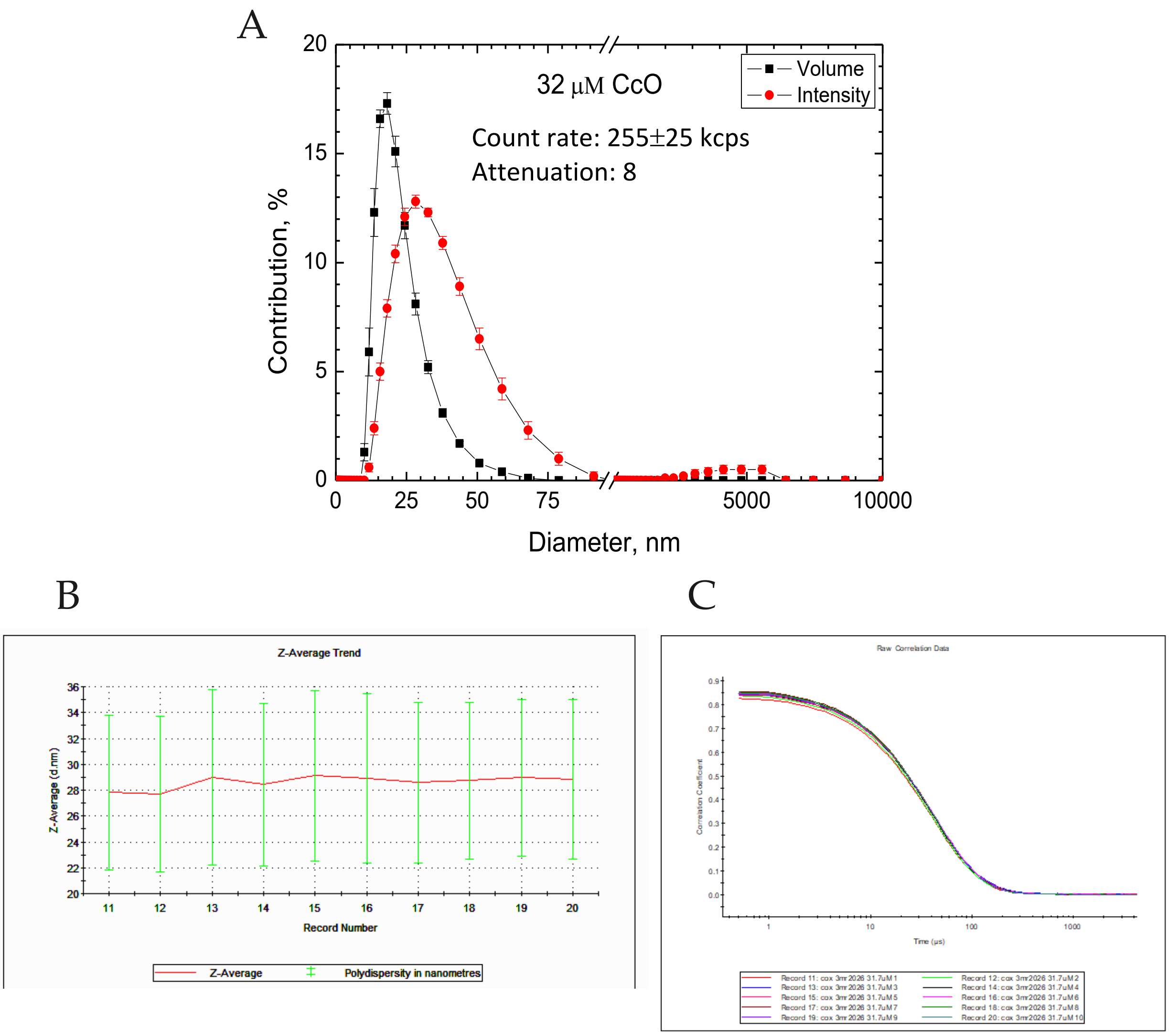

(**D**) – Sample 2. The volume-weighted (black squares) and the intensity-weighted (red circles) distributions are shown together with the count rates and attenuation values.
(**E**) – Sample 2. Z-average trends together with the polydispersity index values.
(**F**) – Sample 2. Correlogramms of the individual measurements.

D

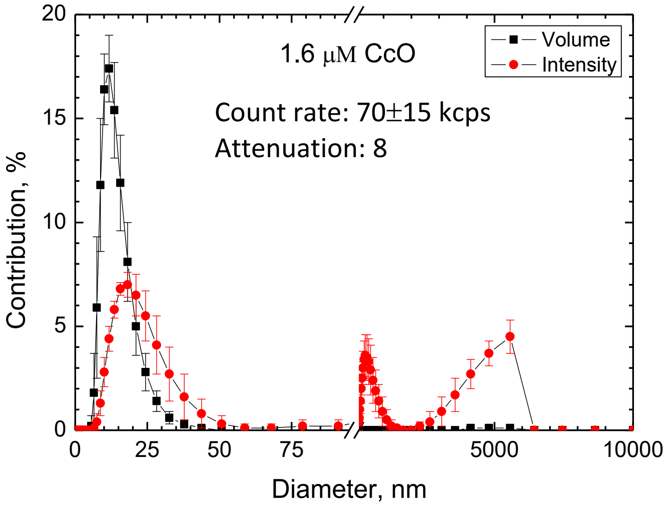

E

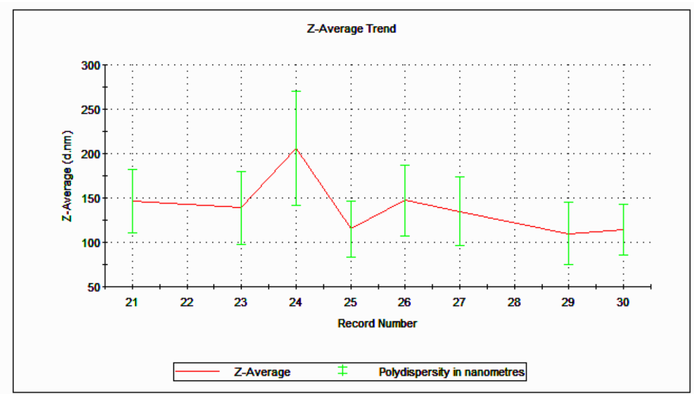

F

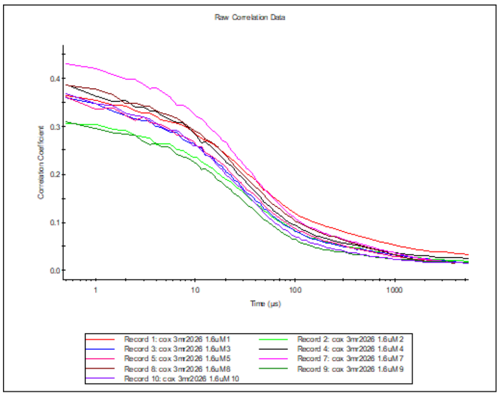

Table S1. Cytochrome oxidase activity of the typical preparations investigated in this work. Activity was assessed by the rate of dissolved oxygen consumption in the presence of the enzyme and a respiratory substrate (see Methods for the details) and is expressed as the enzyme Turnover Number (TN).

**Sample**	**Oxidase activity as TN, e^-/s**
DM solubilized mitochondrial CcO	89±24 (n=5)
DM solubilized *aa_3* oxidase from *R. sphaeroides*	143±15 (n=2)
AmB	120
AmM	104±19 (n=3)
AmD	89
